# Rebound effects in circular business models: A review of cases and mitigation strategies

**DOI:** 10.12688/openreseurope.21309.2

**Published:** 2026-05-07

**Authors:** Ankita Das, Daniel Guzzo, Daniela C. A. Pigosso, Nancy Bocken

**Affiliations:** 1 School of Business and Economics, Maastricht Sustainability Institute, Maastricht University, Maastricht, 6200MD, The Netherlands; 2 Civil and Mechanical Engineering, Section of Design for Sustainability,, Technical University of Denmark, Lyngby, 2800, Denmark; 3 International Institute for Industrial Environmental Economics, Lund University, Lund, 22350, Sweden

**Keywords:** Circular Economy, Circular Business Models, Rebound Effects, Circular Economy Rebounds, Environmental Impact, Business Model Experimentation.

## Abstract

Circular business models are typically designed with the intent to meet customer demands, while resolving environmental issues related to resource usage, product lifetimes and waste reduction. Yet, rebound effects might hinder their positive impacts. Through a systematic literature review of 76 articles, the rebound effects of different circular business model archetypes and suggestions for their mitigation were investigated. The resulting overview unveils the direct, indirect and economy-wide rebound effects of different circular business model archetypes. This is coupled with rebound mitigation suggestions from literature from a business and policy perspective. The findings also revealed an apparent lack of quantitative research on mitigation strategies. The results can be used by researchers to guide research efforts, and by practitioners who want to find ways to mitigate the rebound effects of their circular business models. Future research should focus on creating standardized assessment methods for rebounds and their mitigation strategies.

## 1. Background

Circular business models (CBMs) aim to maximize value by slowing, closing, narrowing and/or regenerating material flows to extend resource use and minimize waste ( Konietzko *et al*., 2020). The implementation of CBMs often involve a number of business model patterns and archetypes, both upstream and downstream ( Pieroni *et al*., 2020; Rosa *et al*., 2019; Salvador *et al*., 2023). While companies experiment with new circular business models to test their desirability, feasibility, and viability, their performance in terms of circularity and sustainability is rarely tested ( Bocken *et al*., 2021a, b). Companies usually rely on rules of thumb to forecast impact (i.e., heuristics related to circular/sustainable design such as guidelines and policies), which can be inaccurate ( Das *et al*., 2022; Pigosso, 2024). Past research has found that negative side effects of circular business models can potentially be prevented during the design and experimentation phase so understanding these is essential ( Das *et al.*, 2023).

Despite the high potential of circular business models to reduce environmental impact ( Fidan *et al*., 2021; Kaddoura *et al*., 2019), rebound effects (REs) are increasingly recognized as a major challenge ( Andrew & Pigosso, 2024; Brockway *et al*., 2021; Zink & Geyer, 2017). Rebound effects are a form of systemic consequence that can offset, or even increase the overall environmental impact ( Zink & Geyer, 2017). While rebound effects can be both positive and negative ( Font Vivanco *et al*., 2022; Zerbino, 2022), the focus of this paper is mainly on negative effects. Prominent examples include increases in consumption as a result of increased efficiency and lower prices ( Chitnis *et al*., 2013), or increased wear and tear on shared products ( Ackermann & Tunn, 2024). These effects arise from changes across business model elements, such as manufacturing and design choices (upstream), consumer behaviour (downstream), and logistics (both upstream and downstream) ( Sarasini *et al*., 2024). Circular business models are by necessity specific to their context and Pieroni et al. (2020) have divided them into seven “archetypal clusters”, meant to reflect the different ways in which circular business models seek to achieve circularity. This points to the importance of analysing rebound effects as they relate to specific circular business model archetypes.

The impact of rebound effects of circular businesses is not fully understood and common forms of measurement like life-cycle assessment (LCA) often fail to account for the impact of rebounds, casting doubt into the true environmental performance of circular strategies ( Lowe *et al*., 2024). Recent research on sustainable actions has shown that rebound effects can undermine potential environmental impact reductions by as much as 50% ( Brockway *et al*., 2021). In the context of mitigating rebound effects, the design of circular business models is an important area of focus as early-stage decisions shape future sustainability performance and limit opportunities for later design adjustments ( Millet *et al*., 2007). Business model innovation is increasingly seen as essential to sustainability ( Bocken & Snihur, 2020), allowing companies to refine models that align with sustainability goals. Failure to assess business model impacts early on increases the risk of unintended consequences and business model lock-in. Managerial decisions in the early design phase can have a big impact on the sustainability performance of a product ( McAloone & Bey, 2009). Pigosso (2024) argues that rebound effects are not yet integrated into the business model design phase and should be proactively addressed by embedding awareness and mitigation strategies from the outset. This aligns with research on product-service systems (PSSs), which highlights how business model design can strongly influence product care ( Ackermann & Tunn, 2024; Müller & Remke, 2025) and sustainability performance ( Siderius & Poldner, 2021; Tukker, 2015), indicating the importance of business model design in managing rebound effects.

The concept of rebound effects dates back to 19 ^th^ century literature on energy efficiency, where it was observed that efficiency gains often led to increased energy consumption ( Jevons, 1866). The “circular rebound effect” specifically was first described by Zink & Geyer (2017) as operating through two primary mechanisms: insufficient substitution and price effects. Insufficient substitution occurs when secondary goods (e.g., recycled or remanufactured products) fail to fully replace goods made from virgin materials. ( Zink & Geyer, 2017). Price effects arise when circular products, being more efficient and cost-effective, lead to monetary savings that are reinvested into additional consumption—potentially more resource-intensive than before ( Zink & Geyer, 2017).

More recent research has identified several further mechanisms through which rebounds can occur and provided a more detailed causal understanding of how rebound effects work ( Castro *et al*., 2022; Guzzo *et al*., 2024; Lange *et al*., 2021). This ranges from economic to behavioral and social practice mechanisms, at both the consumer and producer sides; directly, indirectly, through changes in demand and use intensity on different levels of the economy (on the level of the individual firm, the sector, economy wide or global), and at different points in time (at implementation, in response to implementation or at the point of attempting to mitigate rebounds) ( Aguiar *et al*., 2026; Andrew *et al*., 2026; Ferrante *et al*., 2026; Van Der Loo & Pigosso, 2024). This study uses the term rebound effects (or simply rebounds) instead of circular rebound effects, as many rebound mechanisms apply to circularity as well. An example would be in the classic case of fuel-efficient cars leading to users driving them for longer distances ( Pardi, 2021). Or, in the case of PSS, it has been found that rented items tend to be treated less carefully since consumers feel less attached and bear no long-term responsibility, leading to higher replacement rates ( Ackermann & Tunn, 2024; Schaefers *et al*., 2016). This illustrates a link between business model design and rebound effects ( McKay *et al*., 2025). The shift in ownership and responsibilities in a PSS from the consumer to the producer, which enables renting, is implicated as the cause of careless use, indicating a strong link between business model design and rebound effects. ( Ackermann & Tunn, 2024). The risk of rebound effects of circular businesses is thus that they end up *increasing* overall consumption, rather than reducing it.

While research on identifying and classifying rebound effects is expanding ( Lowe *et al*., 2024; Metic & Pigosso, 2022), practical approaches for identifying and mitigating them during business experimentation remain limited. Studies suggest rebounds can be mitigated through business and policy interventions ( Lowe *et al*., 2024). Some rebounds, such as indirect or system-level effects like re-spending savings from efficiency on other goods and services, may be beyond a single business’s control. However, direct rebounds can be influenced more easily, for example, by providing consumer information or incentive schemes ( Ackermann & Tunn, 2024). Addressing these risks helps businesses meet environmental targets and comply with greenwashing regulations ( Ioannou *et al*., 2022; Robertson *et al*., 2023). Proactively tackling manageable rebound effects can also provide a competitive advantage, demonstrating commitment to sustainability goals ( Schaltegger *et al*., 2012). Meanwhile, indirect and economy-wide rebounds are more effectively addressed through policy ( Lowe *et al*., 2024; Zerbino, 2022). For policymakers, mitigating circular rebound effects ensures that circular economy policies fulfill their goals of reducing waste, conserving resources, and lowering carbon emissions ( Guzzo *et al*., 2025; Zink & Geyer, 2017).

Recent reviews by Lowe *et al*. (2024) and Zerbino (2022) developed methods to manage circular rebound effects, offering general guidelines but highlighting the lack of specific, actionable strategies for circular business models. Das *et al*. (2023) also attempted to classify rebounds across circular strategies through the Circular Rebound Tool, and a set of behavioral design strategies for preventing rebound effects have been consolidated ( McKay & Pigosso, 2025). These studies emphasize the need for a more systematic analysis of rebound effects in circular business models and potential mitigation strategies. While past literature reviews exist, only two articles connect rebound effects to specific circular business model archetypes or explore mitigation through business model design. In their review of case studies Baczyk *et al*. (2024) seek to clarify the role of *consumer behaviour* in shaping the effects of circular business model archetypes once implemented. They also give some suggestions for how rebound effects can perhaps be avoided, although these recommendations take the form of guiding principles and policy recommendations. Another recent contribution to rebound effect mitigation research is Mueller & Remke (2025). Focusing on mitigation through business model design, the authors developed the Circular Systems Sandbox, a business model design tool based on systems and life cycle thinking. The tool helps designers develop circular products and services and to understand the consequences their design choices may have on the environmental impact of their product or service. Although Circular Systems Sandbox connects business model choices to rebound effects, the focus is on the process of business model design, rather than archetype specific rebound effects and how one could mitigate them in practice.

Clarifying the link between specific rebound effects and circular business model archetypes, as well as specific strategies for how to mitigate these rebound effects is an important first step toward integrating rebound mitigation into circular business model innovation, as empirical measurement is often impractical in early design phases. Thus, this paper aims to identify empirically measured rebound effects in circular business models and potential mitigation strategies, addressing the following research questions:

• RQ1: What are the potential rebound effects of different circular business model archetypes?
• RQ2: What strategies have been identified that could mitigate these circular rebound effects?

Section 2 describes the method employed for data collection and analysis. Section 3 showcases the results. Section 4 and Section 5 present the discussion and conclusions of the study.

## 2. Method

**Figure 1.**
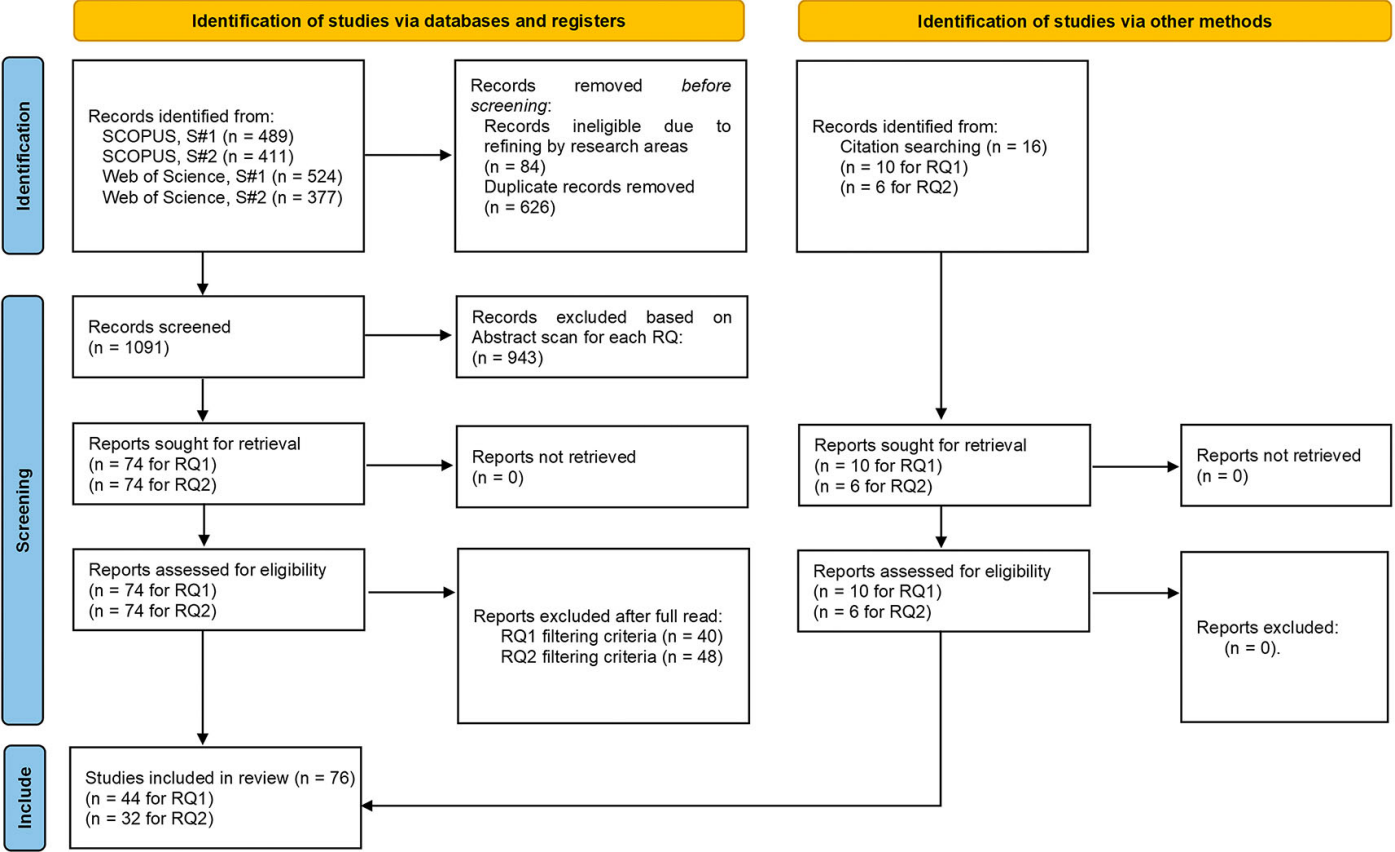
PRISMA flow diagram. (S-1 & S-2 refer to search strings #1 & #2 respectively).

A systematic literature review ( Bryman & Bell, 2011) was conducted to address the research questions, give an overview over the state of the art as well as a direction for future research. This method was chosen to allow a detailed investigation of rebound effects and their mitigation in the context of circular business model archetypes or business model design. This section outlines the systematic review process, including data collection, evaluation, and analysis. Figure 1 illustrates the literature search process and paper selection at each stage. The initial data collection, analysis and coding was conducted by the first author. The studies selected for review and their subsequent coding were periodically independently checked by the other authors for accuracy.

### 2.1. Data collection

Relevant research articles were searched for in Scopus and Web of Science until September 2024 by following the search protocol described in Table 1. The literature search was conducted through two slightly different search strings, which were investigated in conjunction. The first search string included terms that were specific to rebound effects of circular business, while the second search string had a wider focus on rebound effects of business models, while focusing on environmental impact. The primary reason for including two search strings was that while circular business models is a relatively new term, concepts that are included within the term now have existed much longer, such as efficiency and concepts within sustainability, of which circular business models can be said to be subset ( Pieroni et al., 2019, 2020). Further, it was initially found that a focus on just circular and sustainable business models might be too narrow to capture all research available on their related rebound effects. However, broadening the synonyms to include the terms “business model*” and “business*” in S-2 captured too many irrelevant studies, and so this search string was further focused to specifically include environmental impacts. The two search strings were thus used in an attempt to include all articles that might have covered the occurrence of rebound effects in the circular economy before the terms such as circular/circular business started becoming more widely used in academic studies.

**Table 1.** The search protocol followed for the data collection process.

Research Protocol	Description
Search string #1	(“circular economy” OR “circular*” OR “circular business*” OR “sustainable business*”) AND (“rebound effect*” OR “rebound*” OR “unintended consequence*” OR “backfire effect*” OR “take-back effect*” OR “spillover effect*” OR “indirect effect*” OR “secondary effect*”)
Search string #2	(“circular economy” OR “circular*” OR “circular business model*” OR “sustainab*” OR “business model*” OR “business*”) AND (“rebound effect*” OR “rebound*” OR “unintended consequence*” OR “backfire effect*” OR “take-back effect*” OR “spillover effect*” OR “indirect effect*” OR “secondary effect*”) AND (“environment* impact*”)
Refined by Research Areas	Engineering, Environmental Sciences, Ecology, Science Technology, Business Economics, Materials Science, Energy Fuels, Public Environmental Occupational Health, Biodiversity Conservation, Behavioral Sciences, Social Issues, Psychology, Automation Control Systems, Public Administration, Transportation, Communication, Education Educational Research, Telecommunications, History, Philosophy of Science, International Relations, Philosophy, Urban Studies
Scan of	Title, Abstract, Keywords

The initial search returned 1801 articles, which after excluding duplicates and articles of non-relevant research areas was reduced to 1091 articles. The titles and abstracts were then scanned for relevance regarding the two research questions (see Table 2):

• RQ1: The primary inclusion criterion was the clear empirical measurement and identification of rebound effects of circular business models. Articles that simply identified rebound effects but did not show empirical measurement were discarded as they were deemed as not providing enough evidence or explanation for further analysis. This resulted in a total of 74 articles. After a full read, 40 articles were excluded, while 10 new articles were discovered through cross-reference snowballing ( Geissdoerfer *et al*., 2018). This involved scanning the reference lists of initially identified articles for relevant publications that met the filtering criteria. The process continued with snowballed articles until no new relevant publications emerged, resulting in 44 articles.
• RQ2: The inclusion criterion was the identification of strategies for mitigating rebound effects that would be relevant in a business context. This resulted in an initial set of 74 articles. After a full read, 48 articles were excluded and another 6 added through similar cross-reference snowballing as mentioned earlier for RQ1, resulting in a total of 32 articles.

**Table 2.** Filtering criteria for articles selected for review for both research questions.

Filtering criteria for RQ1	Filtering criteria for RQ2
• Study needs to be about rebound effects of circular business models • Study needs to have empirical measurement and identification of rebound effects. For e.g. through regression analysis, scenario-based analysis, attributional/consequential LCAs, system dynamics modeling, etc.	• Suggestions for mitigation strategies for rebound effects of circular business models. • Empirical measurement was not a requirement since rebound effect magnitude quantification was deemed not necessary to devise mitigation strategies, which can be based on qualitative analyses.

The complete list of articles reviewed can be found in Appendix A.

### 2.2. Data evaluation & analysis

The set of articles selected for RQ1 were coded in Excel for qualitative characteristics (see Table 3), to address the nature of rebound effects as highly dependent on the contextual elements, and the boundaries of empirical measurement.

**Table 3.** Coding categories for analyzing final articles.

	Code categories	Definition
1.	Country	Geographical focus of the research study. (e.g., Germany, European Union, OECD countries, USA and Canada, etc.)
2.	Sector	The industry sector(s) the study focuses on. (e.g., mobility, built environment, electrical appliances, etc.)
3.	Data sample size	The size of the data sample used for the empirical research. (e.g., number of case studies, number of study participants, etc.
4.	Data collection & analysis methodology	The type of data collection and analysis methods that were used by the study. (e.g., consequential LCA, system dynamics modeling, regression analysis, etc.)
5.	Circular business model archetypal cluster	The circular business model archetype that is studied by the research article (based on the classification by Pieroni *et al*. ( 2020)).
6.	Rebound effects’ magnitude	The magnitude of the rebound effects empirically calculated and reported by the study.
7.	Type of rebound effect	The category the identified rebound falls into (direct, indirect or economy-wide) in order to determine the potential zone of influence of business and policy.
8.	Rebound mitigation suggestions	Potential rebound mitigation suggestions (e.g., increasing consumer attachment to rental/sharing products, and gamification of usage data to reduce consumption)

The rebound effects identified in the sample were categorized following two lenses, one connected to the circular business model and another to rebound effects.

First, the seven circular business model archetypal clusters defined by Pieroni *et al*. (2020) were used for coding the identified rebound effects in the literature: (1) Dematerialised or efficiency, (2) Collaborative consumption, (3) Product-service systems, (4) Long life, (5) Next life, (6) Circular sourcing, and (7) Circular production & distribution. Although alternative classifications have been proposed (for e.g.: Ellen MacArthur Foundation, 2015; Geissdoerfer *et al*., 2020; Rosa *et al*., 2019; Salvador *et al*., 2023), the Pieroni *et al*. (2020) classification was used because it was found to be the most suitable for our analysis as it most clearly delineates both upstream and downstream circular strategies. In addition, it is based on a wide literature and practice. The upstream/downstream categorization was found to be useful as rebound effects tend to occur due to manufacturing and design choices (upstream), consumer behavior (downstream), and logistics (both upstream & downstream) ( Sarasini *et al*., 2024). Whenever articles reported rebound effects for multiple business model archetypes (for e.g., in a multi-scenario case analysis), the results were split into separate rows corresponding to each circular business model archetype classification.

Second, a widespread categorization of the identified rebound effects into direct, indirect and economy-wide effects ( Greening *et al*., 2000; Sorrell & Dimitropoulos, 2008) was employed. Direct rebounds refer to an immediate increase in consumer demand of the same product/service due to a new circular business model ( Zink & Geyer, 2017). Indirect rebounds refer to increases in demand for other goods and services as a result of a new circular business model intervention ( Zink & Geyer, 2017). And economy-wide rebounds refer to longer-term effects that a new circular business model might have on the market, consumer preferences, societal institutions, regulations, etc. that lead to overall increase in consumption ( Castro *et al*., 2022; Zink & Geyer, 2017). In some cases, it was observed that some articles identified other self-defeating systemic responses that played against the purpose of the circular economy action but were not necessarily rebound effects as per our definition (for e.g., Amasawa *et al*., 2023; Koide *et al*., 2023). At times these were mis-identified in the articles as rebound effects. These were instead classified as unintended consequences and were disregarded from the main analysis.

**Figure 2.**
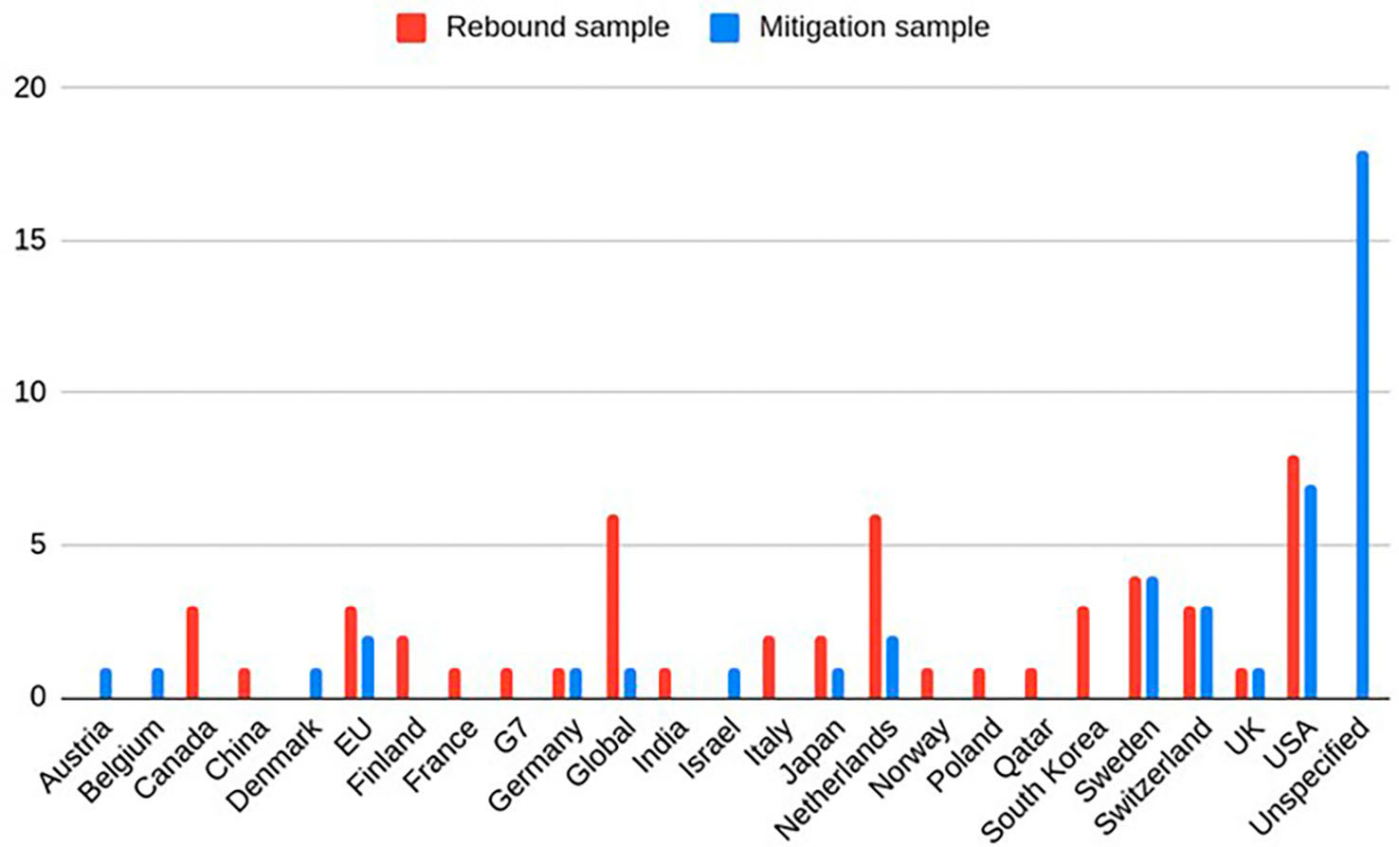
Geographical regions of case studies in rebound and mitigation samples (Global = nonspecific, Unspecified = reviews/conceptual).

The qualitative characteristics of the articles selected for RQ2 were similarly coded in Excel based on Table 3, and their descriptive statistical features were tabulated for graphical presentation ( Figure 2– Figure 4) using Excel. The suggestions for mitigation were then classified into mitigation strategies for business and policy, and further subdivided into ‘measures to reduce consumption’, ‘measures to reduce impact of consumption’ (once the rebound effect occurs), and ‘other measures’. These codes for the rebound effects and mitigation suggestions were then synthesized into broader categories for each business model archetype. This is presented in Table 4 in the Results section. To maximize the traceability of the data the mitigation strategy descriptions have been kept as close to the source as possible, irrespective of their actual potential for the actual mitigation of rebound effects. The complete list of articles reviewed and the corresponding qualitative codes for rebound effects and mitigation strategies identified can be seen in Appendix A and C.

**Table 4.** List of circular business model archetypes, their identified rebound effects and corresponding mitigation solutions.

Circular Business Model Archetype Clusters	Rebound Effect Cases	Rebound Mitigation Suggestions	
		Business	Policy
**Dematerialisation or Efficiency** *(E.g., Dematerialised services, Demand reduction services, Encourage sufficiency)*	**Direct:** None reported	**Measures to reduce consumption:** - Measures to reduce product/service use - Transparency regarding the true environmental footprint of a product or service (e.g., carbon labelling that emphasizes ecological damage of consumption) **Measures to reduce impact of consumption:** - Promote more energy efficient products/services - Pursue regenerative strategies, such as maintaining the health of ecosystems through restoration and remediation, aiming to make a positive environmental impact **Other measures:** - Account for the rebound effect in the impact measurement calculations	**Measures to reduce consumption:** - Urban planning that reduces consumption (e.g., reduce travel to essential services and product) - Incentivize consumers to consistently reduce their consumption on multiple fronts - Increase the cost of energy through taxes **Measures to reduce impact of consumption:** - Decrease cost of labour to promote labour intensive industries **Other measures:** None reported
	**Indirect:** - Increased consumption of other goods and services as a result of reduced usage of a given product/service - Savings re-spent on high impact goods and services		
	**Economy-wide:** None reported		
**Collaborative Consumption** *(E.g., Sharing economy, Co-access, Co-ownership)*	**Direct:** - Increased consumption and product use due to reduced cost and increased convenience	**Measures to reduce consumption:** - Increase opportunities for consumers to feel attached to their shared products - Trial non-economic mechanisms like symbolic rewards, information provision and nudging to reduce rebound effects - Educate consumers about the rebound effects and about the impact of their behavior on the environment **Measures to reduce impact of consumption:** - Shift consumption towards less resource intensive products and services **Other measures:** - Conduct holistic assessment of the impacts to account for rebound effects	**Measures to reduce consumption:** - Better urban planning to incentivize shared products - Policies to increase peer-to-peer sharing - Restrictions on private ownership - Incentivise efforts to change lifestyles of consumers - Reduce consumption of high-emission goods **Measures to reduce impact of consumption:** - Incentivise low-emission technology **Other measures:** None reported
	**Indirect:** - Increase in car passengers, due to ride sharing - Efficiency causing more consumption of other goods and services		
	**Economy-wide:** - Increase in non-operational emissions when consumers switch to another mode of transport - A demand increase in car use would encourage urban sprawl		
**Product-Service Systems** *(E.g., Product-as-a- service, Rental, Hire, Leasing, Pay-per-use, functional sales)*	**Direct:** - Higher replacement rate caused by: ▸ Decrease in product care ▸ Higher consumer expectations regarding the state of the product ▸ Encouragement to use product more - Higher/relevant stock need to meet potential consumer demand) - Greater emotional attachment to second hand clothes means clothes ae discarded less frequently (positive rebound) -Introduction of more fuel-efficient cars results in an increase in travel demand	**Measures to reduce consumption:** - Make efforts to change the lifestyles of consumers towards less impactful consumption - Make users more attached to a product (adapt appearance to user, create product personality) - Use rewards and fines - Design products that feel expensive **Measures to reduce impact of consumption:** - Prevent careless consumption - Inform consumers about the importance of product care - Make timely maintenance easy to access for the consumer - Make the consequence of product care visible to the consumer - Facilitate product care by designing products with automated care functions - Use norms, ratings and other social mechanisms to foster product care - Integrate multiple CE strategies within a business model - Eco-efficient value creation (i.e. do more, with less) - Increased focus on producing from end-of-life input materials - Design to reduce the need for care, and making products more robust to different usage patterns. **Other measures:** - Price control: encourage consumers to pay more for resource efficient solution, so less disposable income is leftover - Conduct LCAs that simulate the effect of the product on system outside of the product system - User and stakeholder involvement in PSS innovation -Design a product that learns the users habits	**Measures to reduce consumption:** - Incentivise reduced consumption of high- emissions goods - Introduce emission trading rules - Energy and carbon tax to reduce the magnitude of rebound effects - Cap and trade schemes that place an upper limit on environmental stress **Measures to reduce impact of consumption:** - Incentivise low emission technology **Other measures:** None reported
	**Indirect:** - Savings spent on higher impact goods and services		
	**Economy-wide:** None reported		
**Long Life** *(E.g., Long life* *products, Products* *with life extension* *services, Reduce, * *Repair, Modular* *design, Refill, * *Upgrading)*	**Direct:** - Repair services may increase sales and consumption	**Measures to reduce consumption:** None reported **Measures to reduce impact of consumption:** None reported **Other measures** - Introduction of a repairability index for products that can indicate a product’s durability	**Measures to reduce consumption:** None reported **Measures to reduce impact of consumption:** None reported **Other measures** - Introduction of a repairability index for products that can indicate a product’s durability
	**Indirect:** None reported		
	**Economy-wide:** None reported		
**Next Life** *(E.g., Direct* *reuse, Next life* *sales, Product* *transformation, * *Refurbish,* *Remanufacture,* *Incentivised return* *& reuse, Recycling, * *Waste Management*)	**Direct:** - Second hand markets could intensify consumption: ▸ By reducing the moral burden of consumption ▸ By allowing frequent buyers of new clothes to easily discard clothes ▸ Decrease intensity of use, as items are resold before their end-of-life - Recycling can increase consumption through moral licensing - Increased consumption from imperfect substitution of primary products - Increased global demand of materials that causes increased energy use	**Measures to reduce consumption:** - Use green design to discourage buying spares - Improve social perception of used or recycled products to displace new products in the market - Only encourage product displacement when the circular system makes this convenient **Measures to reduce impact of consumption:** - Raise awareness about the limitations of recycling, while nudging towards recycling - Facilitate repairs - Work with consumers and policy makers to implement recycle and recovery systems - Design products to allow for consumer upcycling, such as modular products **Other measures**: - Incentivize consumers to spend their money on sustainable goods and services - Evaluate rebound effects relative to the value chain, and solely in terms of emission - Rebound effects should be managed as an operation and supply chain risk - Consider resource specific factors when managing rebound effects	**Measures to reduce consumption:** - Carbon tax - Incentivize consumers to spend their saved money on more sustainable goods and services - Policy to enable battery reuse and support widespread adoption of battery reuse - Discourage primary material consumption, e.g., by reducing the size of garbage bins - Charge unit-based landfill fees **Measures to reduce impact of consumption:** - Work with businesses and consumers to create recycle and recovery systems - Recycled content mandates or taxes on primary consumption **Other measures:** - Implement conservation policies that will protect natural resources
	**Indirect:** - Savings may be spent on high impact goods and services - Second hand clothing markets fail to substitute primary products and increase consumption - Efficiency gains result in increased consumption		
	**Economy-wide:** - Low price of secondhand clothes incentivize increased fashion consumption - Symbiotic rebound; e.g. circular strategies compete for inputs		
**Circular Sourcing** *(E.g., Source* *circular supplies, * *Industrial Symbiosis, * *Renewable energy, * *Using bio-materials)*	**Direct:** - A short supply chain increases sales and consumption	**Measures to reduce consumption:** None reported **Measures to reduce impact of consumption:** - Systemic approach to design to better enable recycling - Pursue regenerative business models **Other measures:** - Maximize the localization of the CE network, and work exclusively with renewable energy - Competitors should collaborate to develop circular infrastructure	**Measures to reduce consumption:** - Implement restrictions on the use of specific products such as fossil-based cars **Measures to reduce impact of consumption:** None reported **Other measures:** None reported
	**Indirect:** - Industrial agglomeration that allows waste minimization creates increased pollution from secondary and tertiary sources		
	**Economy-wide:** None reported		
**Circular** **Production &** **Distribution** *(E.g., Take-back &* *reprocessing used* *products, Cleaner* *production, Eco- * *efficiency, Energy* *efficiency, On* *demand production)*	**Direct:** - Higher efficiency of electrical appliances leads to increased consumption - EV adoption may lead to higher automobile travel demand, due to the positive perception of EVs - Lower cost of Plug-in hybrids result in increased demand - More fuel-efficient cars lead them being driven farther - Convenience and comfort of autonomous vehicles increases travel demand - Car pools may make driving more attractive relative to other modes of transport and increase congestion - More efficient tourism infrastructure leads to increased demand of energy, services & delayed stimulation of regional economic growth - Increased energy efficiency and use of renewable energy led to increased resource use	**Measures to reduce consumption:** - Prevent excessive consumption through sufficiency strategies **Measures to reduce impact of consumption:** - Use additional profit for sustainable innovation - Improve reuse and waste reduction - Ensure that sustainable alternatives are qualitative substitutes **Other measures** - All stakeholders should be encouraged to be part of CE - Managers should develop time-bound tactics to carry out and monitor CE effectively - Market secondary goods the same way primary goods are marketed - Give employees sufficient information, safe working conditions and the right to participation to safeguard working conditions - Use education to overcome stigmas towards secondary goods - Target satiable demand or make efforts to ensure that secondary goods production doesn’t have a big impact on overall prices	**Measures to reduce consumption:** - Efficiency gains can be compensated for with quotas or rationing - Savings from efficiency improvements can be spent in a manner that reduces rebound effects (for e.g. by reducing government debt) - Price control measures **Measures to reduce impact of consumption:** - Create mechanisms for the adoption of more sustainable technologies, when they are ready **Other measures** - Policy incentives that reward investment in mitigation strategies
	**Indirect:** - Energy efficiency improvements and renewable energy use leading to increased production and company growth - Full battery electric cars and fuel cell electric cars have a higher capital cost, reducing overall consumption (positive rebound)		
	**Economy-wide:** - Efficiency gains result in increased economy-wide resource consumption - Insufficient substitution of primary products by circular products, leading to increased consumption - Re-investing effect: increases in profit are re-invested in production factors		
**General** *(Studies not specifically related to any particular circular business model archetype)*	**N/A**	**Measures to reduce consumption:** - Inform and nudge consumer: ▸ Education and raising awareness ▸ Labeling and green labeling ▸ Smart metering and feedback to user ▸ Use default options to guide choice ▸ Guide behaviour through social context **Measures to reduce impact of consumption:** None reported **Other measures:** - Reduce labour productivity to compensate for resource productivity increase	**Measures to reduce consumption:** - Use taxes: ▸ Fuel taxes, carbon taxes or taxes on non- renewable resources, mining taxes and mining royalties ▸ Decrease taxes on labour and renewable resources - Increasing reclamation costs and exploration costs of mining - Include scrap prices on major futures exchanges - Incentives to direct consumption and expenditure **Measures to reduce impact of consumption:** - Fuel efficiency improvements, cap-and trade schemes - Lower the material footprint of all goods and services, e.g. by phasing out environmentally harmful activity **Other measures:** None reported

## 3. Results: Overview of rebound effects of circular business archetypes, and associated mitigation strategies

In the following section the results of the literature review are presented and discussed. First the descriptive characteristics of the papers in the study sample are presented. Second, the primary finding of this study, a classification of commonly measured rebound effects of the seven circular business model archetypal clusters, along with potential mitigation strategies is presented. Then, in the following subsections, the specific findings for each one of the seven circular business model archetypal clusters are discussed.

### 3.1. Descriptive characteristics of the articles in the study sample

**Figure 3.**
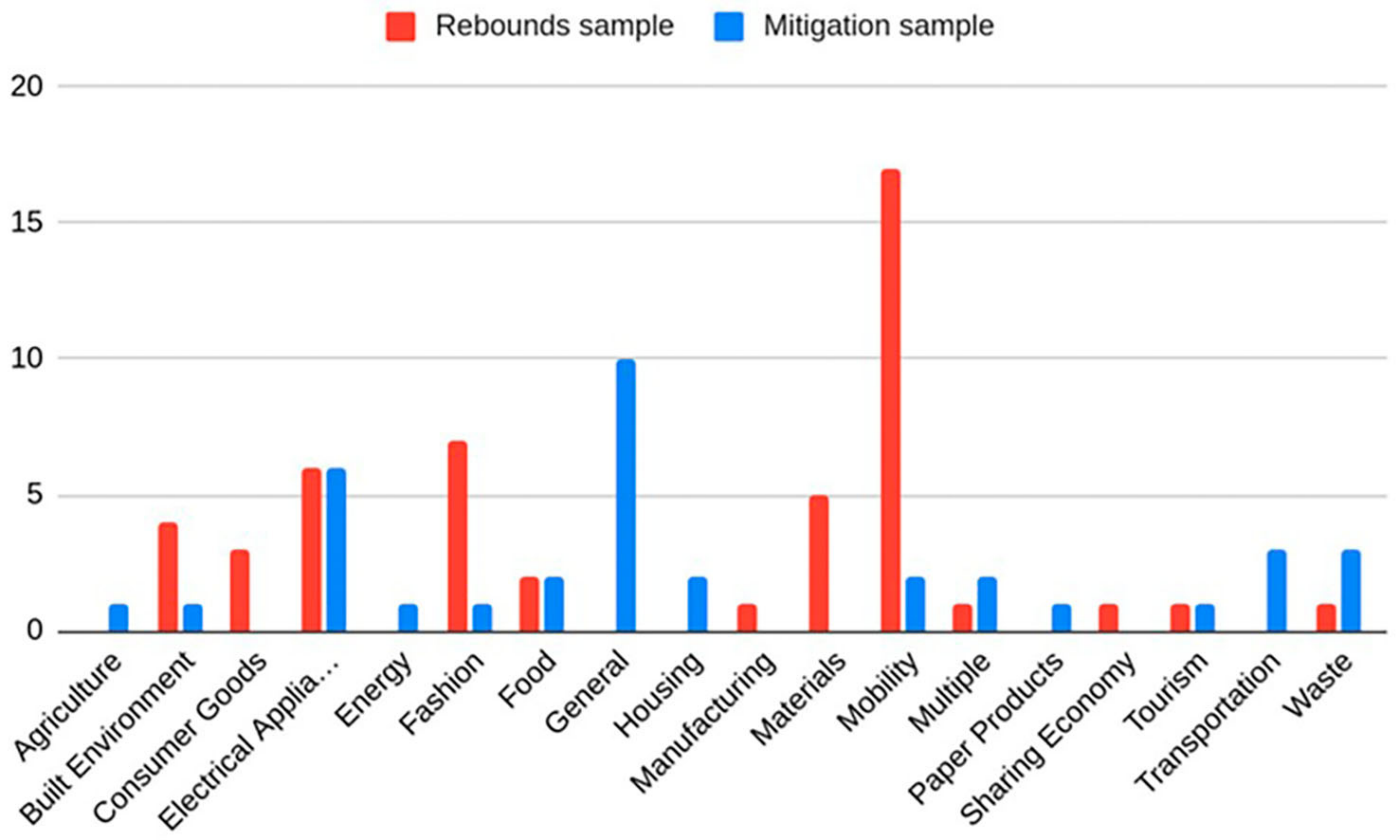
Sectors in rebound and mitigation samples.

**Figure 4.**
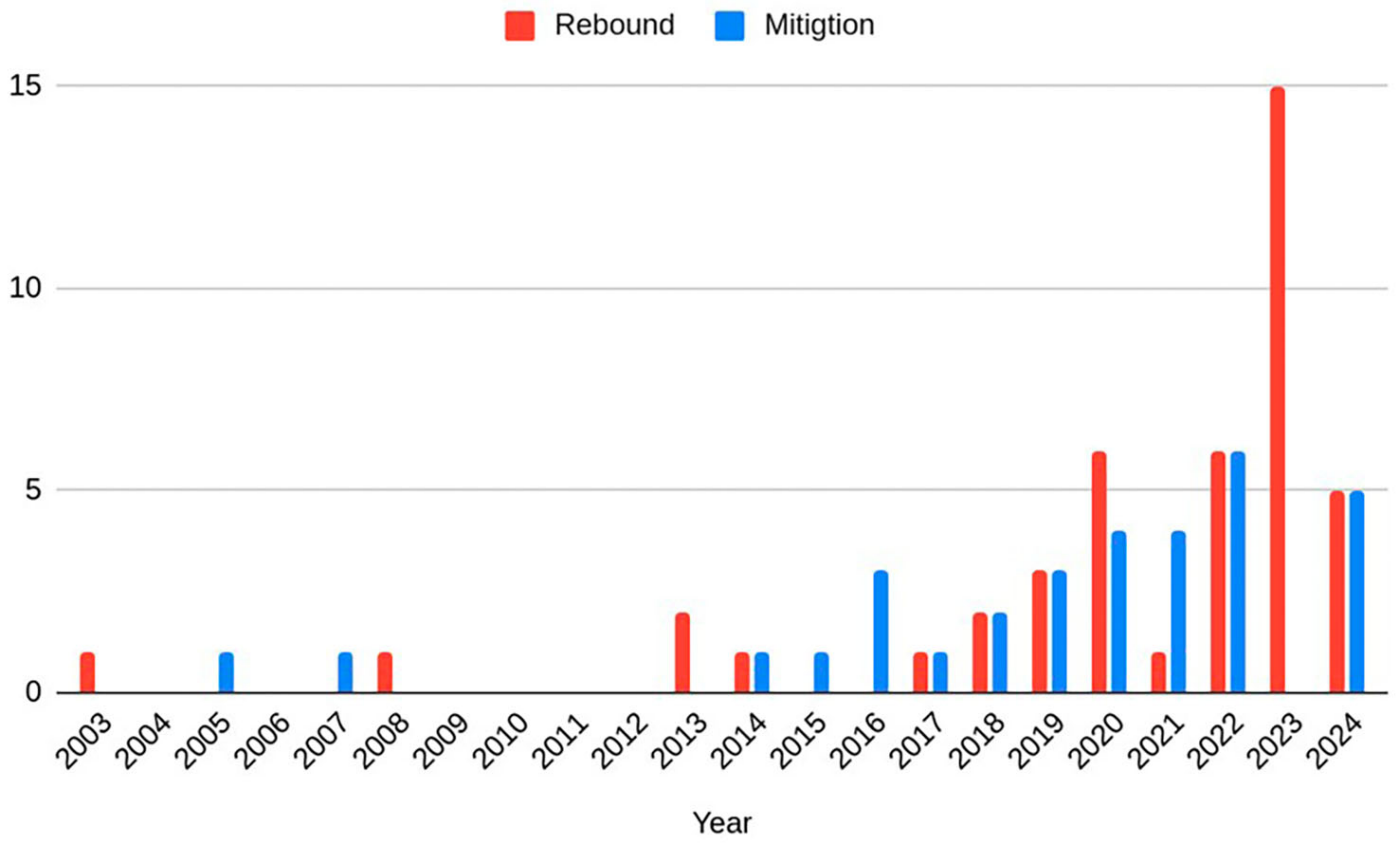
The publication years of the rebound and mitigation papers within the study sample.

The descriptive statistical features of the reviewed studies are illustrated in Figure 2– Figure 4. The regions and sectors of study are presented separately for the two different samples, to reflect that the two different search strings used different inclusion and exclusion criteria. As shown in Figure 2 North America and EU countries were the most frequently studied regions. The “Global” category describes papers where data was not specific to one single region, e.g. from a global market. The “Unspecified” category encompasses papers that conducted literature reviews, or that were not empirical in nature, e.g. conceptual papers. This primarily included papers in the rebounds mitigation sample, as mentioned in Table 2. In the rebound sample the most common sector was mobility, followed by fashion and electrical appliances ( Figure 3). As can be seen in Figure 3, sectors were less clearly defined in the mitigation sample, which included some literature reviews and conceptual papers, with 10 studies not specifying a specific sector. Furthermore, many studies in the mitigation sample were not specific to a given sector (10). In Figure 3, the “Multiple” category refers to a study where data was drawn from many different sectors and the “Sharing Economy” category refers to a study that did not study a sector, but surveyed the sharing economy behaviours of students. The category “General” includes studies that either studied the economy as a whole or studied consumer behaviour itself, rather than any specific sector. Figure 4 shows the year of publication for papers in both samples. The majority of the papers included in this review were published in the 2020’s, with the oldest included papers published in 2003.

### 3.2. Rebound effects of circular business archetypes and associated potential mitigation strategies

The key findings from the systematic literature review are summarized in Table 4. The findings show the potential rebound effects of the seven circular business archetypal clusters identified in the literature, together with potential business and policy mitigation strategies. The extended version of the Table with detailed references can be seen in Appendix B in the Supplementary Information associated with this study. In total, the archetypal clusters with the highest concentration of rebound effects were Circular Production & Distribution (23), followed by Next Life (13) and Collaborative Consumption (12). This might possibly be due to the fact that some of these business model innovations (such as material efficiency, recycling, remanufacturing, etc.) have existed for longer in the business sphere than, for example, sufficiency related strategies. Out of all 76 articles, only 11 lacked mitigation suggestions. However, the effect of these mitigation strategies was often not empirically tested, which limits the understanding of their effectiveness.

The rebound effects and corresponding mitigation suggestions of each of the seven clusters of circular business model archetypes are further explained next with examples.

### 3.3. Dematerialisation or efficiency

The circular business model archetype cluster of ‘Dematerialised or efficiency’ aims to reduce demand for physical products by replacing physical infrastructure with digital infrastructure that stimulates reduced consumption ( Pieroni *et al*., 2020). Research indicated that these strategies were vulnerable to indirect rebound effects, such as price effects, where more efficient services enable increased consumption, either of the primary good or of a secondary good ( Ottelin *et al*., 2017). For example, one study by Walzberg *et al*. (2020) found that energy usage in a smart home - that saved energy through automated temperature regulation - actually used more heating, reducing the amount of energy saved.

Studies suggested that sufficiency strategies that reduce overall consumption could be used more strongly to mitigate these rebounds, alongside even greater efficiency improvements that would lessen the environmental burden of increased use ( Ottelin *et al*., 2017). Others have suggested using ‘carbon labeling’, which would give consumers more accurate information about the true cost of consumption ( Yakobovitch & Grinstein, 2016), or informing consumers about the true environmental footprint of consumption to avoid overconsumption ( MacCutcheon *et al*., 2020). For policy mitigation strategies, some researchers suggested incentivizing decreased consumption ( Sorrell *et al*., 2020), using taxes to increase the cost of energy while simultaneously decreasing the cost of labor, to promote labor intensive industries ( Santarius *et al*., 2023), and utilizing urban planning to reduce travel time ( Ottelin *et al*., 2017).

### 3.4. Collaborative consumption

Business models of the ‘Collaborative consumption’ archetype cluster aim to reduce consumption through the sharing of resources between users, either for free or for a fee ( Pieroni *et al*., 2020). Examples include car sharing or carpooling ( Realini *et al*., 2021), or the sharing of food waste or leftover food ( Meshulam *et al*., 2023).

Several studies indicated that sharing schemes are prone to direct, indirect and economy-wide rebound effects. An example of a direct rebound is increased consumption due to increased convenience or time saved through sharing of resources ( Medina-Tapia & Robusté, 2018; Warmington-Lundström & Laurenti, 2020). One study found evidence of moral licensing and imperfect substitution, meaning that the very act of collaborative consumption encouraged more consumption, while some products also proved to be poor replacements for other products and simply added additional consumption ( Ribera Jemio *et al*., 2024).

Meanwhile the indirect rebounds were often found in the form of price effects, where the savings made from utilizing a sharing platform freed up money that could be used on other goods and services that may have a higher environmental impact ( Meshulam *et al*., 2023; Vélez, 2023; Warmington-Lundström & Laurenti, 2020). One clear example is described by Warmington-Lundström & Laurenti (2020), where people who utilized a boat sharing scheme often spent more money on things like car travel and air travel. On a larger scale, some research has found that cost savings of sharing schemes could cause demand to increase ( Medina-Tapia & Robusté, 2018), while the shift away from one mode of transport to another might cause non-operational emissions to increase, as some vehicles would not be fully utilized ( Amatuni *et al*., 2020).

Mitigation proposals suggested in the literature mainly focused on improving consumer awareness of price effects ( Warmington-Lundström & Laurenti, 2020) and of shifting consumption to goods and services with a lower material footprint ( Vélez, 2023). Alongside these recommendations, measures to increase product attachment, employment of sufficiency strategies to reduce consumption ( Junnila *et al*., 2018) and reducing unnecessary consumption alongside improving the effectiveness of the goods and services has also been proposed ( Harris *et al*., 2021). In terms of policy interventions, it was suggested that urban planning that incentivizes public transport and reduces travel time, alongside restrictions on private transport and policies that support peer-to-peer sharing, could help mitigate rebound effects ( Medina-Tapia & Robusté, 2018). Furthermore, it was suggested that policy could help steer consumption towards goods with a lower footprint and help create mechanisms that support the adoption of new and more sustainable technology ( Vélez, 2023).

### 3.5. Product-service systems

The archetype cluster of “product-service systems” serves as an umbrella term for combinations of products and services such as product-as-a- service, rental, hire, leasing, p-per-use and functional sales ( Pieroni *et al*., 2020; Tukker, 2004), that aim to reduce consumption by offering products as services.

A common direct rebound that was found in the research on PSS has been careless consumption ( Tunn & Ackermann, 2020), the tendency to be careless with objects that one does not own. Research has also indicated that if consumers expect good-as-new products, or if consumers are encouraged to use products more, then consumption might increase in a PSS ( Tunn & Ackermann, 2020). Research has also indicated a risk that as consumers are encouraged to consume more, in order to increase profits, higher stocks would need to be maintained to manage spikes in consumption which leads to increased environmental impact ( Metic *et al*., 2024). Similar to collaborative consumption, research indicated that indirect rebound could occur if consumers save money from engaging in a PSS and then spend that money on other goods and services ( Vélez, 2023).

To decrease the effect of careless consumption, it has been suggested that products should be designed for care ( Tunn & Ackermann, 2020) and to increase the consumers attachment to the goods ( Junge, 2021). It has also been suggested that careless use should be penalized ( Tunn & Ackermann, 2020). If the price of the goods could also be controlled, that could mitigate price effects ( Kjaer *et al*., 2019; Laurenti *et al*., 2016). Other suggestions involved incentivising/promoting a change in the lifestyle of consumers towards less resource intensive products and services (for e.g., by making less impactful consumption more desirable or convenient or nudging towards more careful consumption) ( Vélez, 2023). Additional strategies included monitoring or forecasting the effects of the business model using LCA ( Abdoli *et al*., 2019; Alfarisi *et al*. 2023; Koide *et al*., 2022) or to be more efficient in value creation and use more recycled input materials ( Alfarisi *et al*. 2023). Furthermore, policy could incentivize low emission technology as well as reduced consumption of high emission goods, making price effects less pronounced ( Vélez, 2023). Other authors have suggested improving emission trading rules and incentivizing public transport in the mobility sector ( Abdoli *et al*. 2019), or the implementation of energy or carbon taxes, along with cap and trade schemes to reduce the magnitude of rebound effects and limit environmental damage ( Alfarisi *et al.*, 2023).

### 3.6. Long life

The archetype cluster “long life” refers to business models focused on extended product lifetimes for the same user often facilitated by design for attachment or timeless design, durability, and high levels of warranties and service, coupled with a premium pricing model ( Bocken *et al*., 2016). Despite relatively fewer empirical studies for this archetype, Beulque *et al*. (2023) found that offering repair subscription services can cause more sales, and a higher purchase frequency, resulting in a direct rebound effect. In the case of modular devices, one concern has been the possibility of over provisioning of modules that could lead to increased consumption ( Proske & Jaeger-Erben, 2019).

In order to mitigate rebound effects of “long life” business models, studies proposed that a repairability and durability index could be introduced to support consumer decision making by making the repairability and durability of a product known, thus supporting sufficiency and reducing consumption by steering consumption towards more durable and repairable products ( Beulque *et al*., 2023).

### 3.7. Next life

The circular business model archetype cluster “next life” refers to business models that help extend the lifetime of products and to slow resource loops. While long life aims to extend product life time for a single user, next life aims to extend the material or product lifetime beyond the single user. This can be facilitated by strategies such as design for dis-and reassembly, upgradability, maintenance and repair, as well as product take-back models and second hand platforms ( Bocken *et al*., 2016; Bocken & Short, 2020).

Studies have indicated that second hand markets could *increase* consumption, as consumers simply consume more ( André & Björklund, 2023; Carrasco Campos, 2022), as the reduced price makes increased consumption possible, and the lower quality increases the need for additional consumption ( André, 2024). Some studies have found that recycling can provide moral licensing to incentivize additional consumption, since consumers can view the recycled alternatives as more eco-friendly and thus end up using more of the products, increasing overall consumption related impacts ( Catlin & Wang, 2013; Maier *et al*., 2023). As with many other archetypes, next life strategies were found to be prone to price effects ( André, 2024; Makov & Font Vivanco, 2018; Wiprächtiger *et al*., 2022). It is also important to note the limit of recycling strategies. Recycle, remanufacturing and reuse strategies have been found to lead to imperfect substitution ( Makov & Font Vivanco, 2018; Safarzynska *et al*., 2023; Sourabh *et al*., 2024). Furthermore, on an economy-wide scale, next life strategies may be prone to what Figge & Thorpe (2019) calls a symbiotic rebound, where choosing one CE strategy may move us away from another (perhaps more resource effective) strategy. This involves shifting to biocentric and biomimetic design, identifying environmental degradation and taking steps to revert it in the design and development phase in order to create nature-positive business models ( de Souza *et al*., 2019).

As for mitigation, researchers have pointed to the need to raise awareness about the real effects of recycling and its limitations, as well as to promote behavior change amongst consumers to stop overconsumption ( Catlin & Wang, 2013; Makov & Font Vivanco, 2018). Other suggestions were to improve the social perception of secondhand goods and to facilitate repairs, to improve substitutability ( Makov & Font Vivanco, 2018). For policy, researchers have suggested measures such as a carbon tax, steering consumption ( Carrasco Campos, 2022) and supporting recycling through cooperation with the private sector and consumers ( Dzombak *et al*., 2019).

### 3.8. Circular sourcing

The archetype cluster “circular sourcing” refers to upstream business model strategies like industrial symbiosis or closed-loop production that aim to create value through use of recycled materials, byproducts, and waste as inputs in their new products or services ( Pieroni *et al*., 2020).

Research has indicated that circular sourcing could be prone to direct rebound effects, such as industrial agglomeration *increasing* local pollution ( Fons *et al*., 2003). Meanwhile a shorter supply chain means more efficiency, which in turn can cause consumption to increase as prices go down ( Vegter *et al*., 2023). Other authors have found that the perception of Electric Vehicles (EVs) as a ‘green’ alternative could lead to increased driving, as opposed to using more efficient modes of transportation (e.g. public transportation) ( Kosmidis *et al*., 2023). As for indirect rebound effects, Fons *et al*. (2003), suggested that strategies such as waste minimization risked leading to greater local pollution as the required waste management systems would incentivize geographical business concentration, which in turn attracts other business.

Limited direct mitigation suggestions were found in the literature connected with circular sourcing. However some authors suggested that such rebound effects where gains can be lost due to increased consumption can be counteracted by implementing regenerative strategies ( de Souza *et al*., 2019).

### 3.9. Circular production and distribution

The archetype cluster “circular production and distribution” refers to upstream business model strategies that can, for example, involve producing goods and services on demand to reduce overconsumption, reducing waste and pollution by using cleaner energy/employing efficiency strategies, or closing the material loop through take back schemes or other forms of reprocessing of goods ( Pieroni *et al*., 2020).

In the case of strategies that aim to increase efficiency or reduce consumption, some studies found that these strategies can result in direct or indirect rebounds. For example, Konash & Nasr (2022) describe a direct rebound in a case study of a medium sized complex parts manufacturing company from New York, USA. They found that the company’s circular efficiency measures, such as equipment upgrades, energy and water use monitoring, optimizing operating schedules, recycling water and materials, reducing waste, performance improvement programs, actually led to increased resource use in the short term and a clear backfire effect. Energy consumption did however decrease over the long term but with direct and indirect rebound effects. Skelton *et al*. (2020) also pointed to this risk of increased consumption on a greater scale if efficiency resulted in reduced material input prices. In a similar vein, van Laarhoven (2023) suggested that if increased profit due to efficiency gains was reinvested in production factors, this could lead to increased consumption.

There were many suggestions for mitigation in the literature. Most prominent perhaps were suggestions to raise awareness among consumers ( Kagawa *et al*., 2013; Shinde *et al*., 2022), and attempt to steer spending away from goods and services with a greater material footprint ( Kawajiri *et al*., 2015; Throne-Holst *et al*., 2007). Alongside these suggestions were suggestions for the adoption of renewable energy and cleaner technology ( Onat *et al*., 2023). At a policy level, it has been suggested that prices be controlled, and to support the adoption of newer, more sustainable technologies ( Guzzo *et al*., 2023; Lifset *et al*., 2024). Additionally, in the specific case of electric vehicle (EV) adoption, Kosmidis *et al*. (2023) have suggested that restricting the use of cars could mitigate the effect of consumers choosing EV’s ahead of public transport alternatives.

### 3.10. General mitigation suggestions

Some mitigation suggestions were identified that were not related to any specific circular business model archetypal cluster, but instead were suggested as mitigation of rebound effects in circular business in general. These suggestions were perceived as relevant and are included in a final row in Table 4. One example of such a mitigation strategy was raising consumer awareness in various ways, through education, labeling or using technology to provide feedback to the consumer, to make them more aware of their consumption ( Lowe *et al*., 2024; Ottelin *et al*., 2020; Polizzi di Sorrentino *et al*., 2016). Walnum *et al*. (2014) suggested that rebound effects could be mitigated if efficiency improvements were used for something other than reduced prices or increased production, such as reduced working hours.

For policy, the general suggestions for mitigation were primarily to use various tax incentives to manage rebound effects ( Lowe *et al*., 2024; Ottelin *et al*., 2020; Walnum *et al*., 2014). The primary purpose of the suggested taxes was to increase the cost of environmentally harmful consumption, such as taxing non-renewable resources ( Ottelin *et al*., 2020), carbon or mining ( Lowe *et al*., 2024), although Ottelin *et al*. (2020) also suggested decreasing taxes on labor and renewable resources in order to favor renewable resources and business sectors that are labor intensive as opposed to material intense.

## 4. Discussion

In this section the primary findings of the systematic literature review of rebound effects on the seven circular business model archetypal clusters, and their potential mitigation strategies are discussed, followed by advice for practitioners and the limitations of the study.

### 4.1. Rebound effects of circular business models

The main finding of this study is that although rebound effects significantly impact circular economy strategies, there is little structured knowledge to guide practitioners in implementing circular business models. The sample of 44 empirical studies showed rebound effects in every circular business model archetype, suggesting they may be an inherent aspect of circular models. These effects arise through a variety of mechanisms when attempting to slow, close, or narrow resource loops which include price effects, careless consumption, substitution effects, cross-rebound effects ( Font Vivanco *et al*. 2022), as well as consumer perception of products and their environmental impact and moral licensing. The findings revealed significant overlap in rebound effects across circular business model archetype clusters, particularly regarding efficiency gains and cost reductions leading to increased consumption. This is expected, as efficiency drives circularity ( Blomsma *et al*., 2019; Zink & Geyer, 2017), though each business model achieves it differently. Some rebound effects are model-specific, such as careless consumption undermining product-service systems ( Junge, 2021; Tunn & Ackermann, 2020), moral licensing encouraging consumption in next-life models ( André & Björklund, 2023; Carrasco Campos, 2022; Ciechelska *et al*., 2023) and modal shifts in collaborative vehicle consumption ( Amatuni *et al*., 2020; Realini *et al*., 2021). The results highlight the complexity of rebound effects, particularly their interaction with consumer behavior.

With regard to RQ2 the aim of this study was to provide insights into rebound effects of specific circular business models and the possible ways to mitigate them, that could be useful for future research as well as practitioners. Many mitigation strategies focus on influencing consumer actions through laws, incentives, nudging, price controls, or information campaigns ( Carrasco Campos, 2022; Kjaer *et al*., 2019; Laurenti *et al*., 2016; Sorrell *et al*., 2020). It is clear from these examples that circular businesses need consumer participation to transition from linear to circular consumption. However, there was little discussion on rebound effects linked to producer-producer business model relationships. And, while many rebound mitigation strategies were suggested, they were rarely empirically tested to assess their effectiveness and thus their actual effects remain theoretical. Indeed, only 32 of 45 papers linked mitigation strategies to specific rebound effects. Additionally, some suggested strategies were overly general and could unintentionally reinforce rebound mechanisms, increasing environmental impact. For example, improving fuel efficiency to reduce environmental impact ( Onat *et al*., 2023) may be counterproductive, as efficiency gains often trigger rebound effects. This uncertainty highlights the need for further quantitative empirical research to determine which mitigation strategies are effective, under what conditions, and how different circular strategies can be combined to minimize rebound risks.

Additionally, the literature sometimes used “rebound effects” more broadly to describe unintended consequences of circular business models. While rebound effects typically refer to negative outcomes linked to market dynamics and consumer behavior, unintended consequences encompass broader, unforeseen impacts of implementing such models. Business decision-makers may not consciously distinguish between induced impacts, unintended consequences, and rebound effects. In at least 13 papers from the initial sample, this distinction was unclear. We argue that “rebound effects” should specifically refer to systemic or behavioral responses triggered by adopting a circular business model, that are not essential elements that are part of its implementation ( Kjaer *et al*., 2018). This is important since without a clear understanding of how rebound effects arise or how they differ from unintended consequences, measurement and mitigation efforts may fall short ( Friedrichsmeier & Matthies, 2015; Guzzo *et al*., 2024).

Further, this paper aims to provide insights into rebound effects in specific circular business models and potential mitigation strategies useful for research and practice. Organizing rebound effects by circular business model archetypes, assuming they are shaped by the model implemented, offers clearer information on risks and mitigation strategies applicable to specific businesses. Similarly, categorizing rebound effects as direct, indirect, or economy-wide helps assess how innovations impact different economic levels and interact. Identifying the system in which a rebound effect occurs may better enable practitioners and policymakers to influence environmental impacts. Additionally, the systematic overview can help determine which rebound effects and mitigation strategies fall within the zone of influence of practitioners, hopefully further aiding them in developing strategies to avoid or mitigate rebound effects. Finally, such an overview may offer valuable guidance in early business model design, where companies often rely on “rules of thumb” rather than detailed assessments to estimate environmental impacts ( Das *et al*., 2022; Das *et al*., 2023).

Past systematic reviews of circular economy rebounds indicate limited awareness of the topic in practice ( Lowe *et al*., 2024; Zerbino, 2022). In their review of rebound estimation methods Lowe *et al*. (2024) found that beyond tax and pricing policies, discussions on mitigating rebounds are scarce, especially in a circular economy context. They emphasized the need for ex-ante planning and measurement in business experimentation. Meanwhile, Baczyk *et al*. (2024) explored the rebound effects with a focus on consumer behaviour, suggesting how rebound effects from consumer behaviour might be mitigated, however the suggestions are general guiding principles of mitigation, rather than strategies designed to fit specific scenarios. Indeed, past mitigation strategies have been general, vague, and more difficult to convert into practice ( Zerbino, 2022), which also applies to many mitigation strategies in this review. Further research is needed to refine mitigation strategies, but categorizing them into business- and policy-focused approaches could help practitioners identify what falls within their influence. This would guide context-specific actions and integrate awareness of rebound effects into business model design. This is especially important as Das *et al*. (2022) found that small and medium-sized business practitioners lack structured knowledge to support low-impact design decisions. Findings also suggest that coordinating business and government efforts could strengthen mitigation by aligning strategies for greater impact.

Lastly, past research calls for treating rebound effects as a risk to be managed in circular strategies ( Zerbino, 2022). Meanwhile, extensive studies have explored the circular economy’s role in enhancing social sustainability and addressing the needs of disadvantaged communities ( Mies & Gold, 2021; Schröder *et al*., 2019). For example, some suggested mitigation strategies, e.g. price controls, suggest addressing rebound effects by making consumption more expensive. However, while raising prices may reduce consumption, it places disproportionate responsibility on lower-income groups, potentially exacerbating inequality ( Mies & Gold, 2021; Schröder *et al*., 2019). Such approaches may also backfire if people simply switch to cheaper, more environmentally harmful products, and could fuel class-based resentment toward circular solutions perceived as accessible only to the wealthy. In some cases, accepting low rebound effects may be necessary ( Font Vivanco *et al*., 2022). For instance, poorer households requiring thermal retrofits to escape energy poverty would generate significant energy rebounds but are essential for a dignified life ( Bouzarovski *et al*., 2017). Findings from this research can help inform practitioners and policymakers about rebound risks while considering social benefits. However, concerns about rebound effects should not deter circular strategies, and unavoidable rebounds should be weighed against their potential social impact.

### 4.2. Contributions to research, practice and policy

Although previous reviews of circular rebound effects have been conducted (e.g., Brockway *et al*., 2021; Font Vivanco *et al*., 2022; Lowe *et al*., 2024; Metic & Pigosso, 2022; Sorrell *et al*., 2020; Warmington-Lundström & Laurenti, 2020; Zerbino, 2022), this is the first to examine them in the context of circular business model archetypes. Business models significantly influence material flows and energy use ( Bocken *et al*., 2016; Halme *et al*., 2007), making it crucial for researchers and practitioners to understand the underlying rebound effects, to maximize the benefits of circular business models. One of the main contributions of this review is systematically summarizing current research on circular business models and rebound effects, serving as a foundation for future studies. The second major contribution is consolidating rebound mitigation strategies suggested by literature. Most of these suggestions are yet to be empirically tested. Mapping these suggestions to specific business model archetypes, provides a starting point for further research on their effectiveness. The findings highlight knowledge gaps and existing conclusions, guiding future investigations and informing businesses and policymakers on best practices to optimize circular model benefits.

The findings of this research can be useful to decision-makers navigating the transition to circular business models, specifically to addressing uncertainties around sustainability gains being offset by rebound effects. The systematic overview presented by this study can also support policymakers in implementing circular economy policies while considering associated risks, especially economy-wide rebound effects. For practitioners the results provide suggestions for mitigation strategies that can be trialed and refined. Innovators can use these insights to identify and address potential rebound effects early in business model design, reducing risk—especially in light of potential new regulations. Designers can also consult the overview during the early ideation phase of business model design ( Bocken & Snihur, 2020) to assess and mitigate rebound effects. Further, in the experimentation phase, the systematic overview can continue to be a reference for identifying rebound effects and refining mitigation strategies as business model performance is evaluated and adjusted.

### 4.3. Limitations and future research

This study had some limitations. First, there are limitations pertaining to the chosen method. The literature search was carried out in only two databases, this might have led to search results being more limited than they otherwise might have been. Additionally, although the title, abstract and full text scans were carried out systematically, there is a chance for inadvertent omissions of relevant data, due to human error. It is also worth noting that the search was carried out in late 2024 and for that reason it does not encompass all the more recent contributions to the field. Further, most of the business case study examples found in the literature were from Europe or North American, limiting generalizability of results. As noted by Baczyk *et al*. (2024), consumer behavior is often specific to a certain place and culture and it’s therefore important to always encourage the development of circular strategies that are tailored to the context within which the business is embedded.

Second, the non-identification of rebound effects in a given archetype does not mean they do not exist. Some circular business models are more prevalent and researched than others, for example, the ‘Circular Production & Distribution’ and ‘Next Life’ archetypes had more research studies compared to other archetypes. More research should be directed towards understanding rebound effects in the less well researched circular business archetypes. Additionally, the findings must be considered within the boundaries set by the primary studies from which the rebound effects and mitigation strategies were extracted. For example, a study on rebound effects of production of remanufactured marble did not account for the savings from avoiding virgin raw material extraction ( Zerbino, 2022). The researchers note this limitation as “the capacity to preserve mountains from anthropogenic activity cannot be easily translated into saved emissions” ( Zerbino, 2022, p.9). So, while some activities might appear to generate rebound effects, they might have long-term positive societal or natural restorative or regenerative effects, that are hard to quantify, within impact measurement boundaries.

Third, while several mitigation strategies were identified, many were broad suggestions rather than actionable steps (for e.g., in the case of PSS, price controls and steering consumers towards less impactful consumption or measuring impacts of change). While others referred to initiatives that themselves can also lead to rebound effects (e.g., suggesting to improve the social perception of secondhand goods, practice that itself is connected to several rebound effects). Further, some of the suggested policy-oriented strategies, such as urban planning, fuel or carbon tax and restrictions on fossil fuel transportation are not specific to the circular economy, and could lead to additional rebound effects if not carefully planned and implemented. Moreover, most strategies were not empirically tested, meaning their actual effectiveness remains uncertain. This study, therefore, relies on mitigation strategies as reported by authors, highlighting the need for future research to identify credible, context-specific strategies and quantitatively assess their effectiveness.

Overall, while understanding rebound effects and mitigation strategies might be complex due to their context-specific nature within circular business models, more research is needed to experiment with these strategies through modeling, scenario analysis, and real-world testing. Accumulated knowledge could lead to standardized procedures for measuring and mitigating rebound effects by design while accounting for environmental impact variations. Institutional analyses considering norms, values, culture, and legislation ( Scott, 2013) could also be beneficial. In spite of this lack of knowledge on how to most effectively mitigate rebound effects, there are some early signs of companies looking to gain insight into how rebound effects can potentially occur in their new business model offerings: such as the outdoor-goods manufacturer VAUDE, conducting a LCA study to evaluate the environmental impact of different scenarios before adopting a PSS business model ( Friedrich, 2023).

## 5. Conclusion

While research on circular rebound effects has grown, there has been a lack of organized knowledge on mitigating these effects within business models. This study aimed to investigate (1) the potential rebound effects of circular business model archetypes and (2) identified strategies for their mitigation through a systematic literature review. It provides a structured overview mapping rebound effects across seven circular business model archetypal clusters, and suggestions for ways to potentially mitigate them. The study highlights where empirical research has focused on direct, indirect, and economy-wide rebound effects and where future research opportunities exist. It also provides policymakers and practitioners with a systematic overview of risks associated with circular business models and potential mitigation strategies. However, while mitigation strategies have been suggested, they remain largely untested, and their effectiveness is uncertain.

The results indicate the need for more research to attribute rebound effects to specific circular business model choices, standardise methods to analyze the behavior and magnitude of these rebound effects, and develop mitigation strategies. Additional research could also refine the key findings of this paper ( Table 4) into a practical tool or process that could enhance their usability.

Finally, while mitigating rebounds is crucial, their occurrence must be weighed against the broader societal benefits of circular business models. Given the limited research on effective mitigation strategies, practitioners must carefully evaluate the impact of circular business models to ensure they achieve their intended sustainability goals. Identifying and addressing rebound effects is challenging but essential, and requires more research efforts.

### Ethics and consent

Ethical approval and consent were not required.

### Data availability statement

#### Underlying data

All data underlying the results are available as part of the article and no additional source data are required. A review protocol was not registered prior to the start of the review.

#### Extended data

Data sample records: Rebound effects in circular business models: A review of cases and mitigation strategies. Zenodo. https://doi.org/10.5281/zenodo.16985825 (Das, 2025).

The project contains the following data:

• List of papers selected for review
• List of circular business model archetypes, and identified rebound effects & mitigation strategies with detailed references
• Coding of rebound effects and potential mitigation strategies of circular business model archetypes
• PRISMA checklist

#### Reporting guidelines

PRISMA checklist for ‘Rebound effects in circular business models: A review of cases and mitigation strategies.’ Zenodo. https://doi.org/10.5281/zenodo.19700219 ( Das, 2026).

Data are available under the terms of the Creative Commons Attribution 4.0 International license (CC-BY 4.0).
