# Supplementary material for: Rebound effects in circular business models: A review of cases and mitigation strategies
Source: Open Res Eur. 2026 May 7;5:314. Originally published 2025 Oct 15. [Version 2] doi: 10.12688/openreseurope.21309.2 (PMC13231136; doi:10.12688/openreseurope.21309.2)
Supplement: Peer review history

# Reviewer report: Reviewer response for version 2

**DOI:** 10.21956/openreseurope.24781.r74513

**Published:** 2026-06-13

**Authors:** Changhee Kim

**Affiliations:** 1 Xi'an Jiaotong-Liverpool University, Suzhou, China

Thank you for the opportunity to review the revised manuscript, *“Rebound effects in circular business models: A review of cases and mitigation strategies.”*

 The authors have made commendable efforts to address the initial feedback, particularly by expanding the discussion of practical implications and attempting to clarify key concepts. However, despite these additions, some structural inconsistencies, methodological ambiguities, and logical gaps remain unresolved.

 Further clarification and correction are therefore needed before the manuscript can be fully approved. That said, I genuinely enjoyed reading the article, and I would like to thank the authors once again for their valuable contribution to the circular business model discipline.

**Comment #1:**

 On page 8, the authors suggest that the higher concentration of rebound effects observed in the Circular Production & Distribution, Next Life, and Collaborative Consumption archetypes is *“possibly be due to the fact that some of these business model innovations (such as material efficiency, recycling, remanufacturing, etc.) have existed for longer in the business sphere”.* However, this explanation is not empirically tested or supported anywhere in the manuscript. Figure 4 presents publication years only for the full sample, without disaggregation by archetype, so no data is provided that directly substantiates this interpretation.

 Moreover, at least one equally plausible competing explanation is not considered. These archetypes may attract more rebound studies not because they have existed for longer, but because their rebound mechanisms are more visible and more amenable to empirical measurement (e.g. demand increases or consumption changes driven by price effects). Presenting only a single post‑hoc interpretation, without acknowledging alternative explanations or providing the data needed to assess it, may give readers a stronger impression than the results actually justify.

 Therefore, the authors should either: (a) provide disaggregated data (e.g. publication years by archetype) to support their interpretation, or (b) reframe the explanation as one of several competing hypotheses and present it as a potential direction for future research rather than an explanatory conclusion.

**Comment #2:**

 The manuscript reports a total of 76 papers as the review sample, composed of two separate subsamples: 44 papers selected for RQ1 and 32 papers selected for RQ2. The PRISMA diagram in Figure 1 also sums these precisely to 76 (44 + 32 = 76), which suggests that there is no overlap between the two subsamples. However, the main text does not explicitly confirm whether the two samples are mutually exclusive, and given that both samples were derived from the same initial pool of 74 papers using the same search terms, no explanation is provided for how this complete separation was achieved.

 This ambiguity is important for two reasons. First, it is analytically plausible and quite common for studies that empirically measure rebound effects (RQ1) to also propose mitigation strategies (RQ2), since empirical work often leads to policy or practical implications. If a substantial number of papers appear in both samples, it becomes difficult to regard the two research questions as being addressed by independent sets of evidence, and the total sample size of 76 may give an exaggerated impression of the diversity of the actual evidence base.

 Second, the statement on page 8 that *“out of all 76 articles, only 11 lacked mitigation suggestions” *assumes that all 76 papers form a single integrated unit of analysis, which is only valid if any overlap between the two subsamples has been clearly confirmed. At present, this assumption remains unverified. (= If only 11 papers lacked mitigation suggestions, it logically follows that 65 papers (76-11) actually contained mitigation content?)

 Therefore, the authors should clarify whether there is any overlap between the RQ1 and RQ2 samples. If overlap exists, its extent should be specified, along with an explanation of how it was handled in the analysis design. If the two samples are indeed mutually exclusive, a brief methodological explanation of how such separation was possible from the same initial literature pool should be provided.

**Comment #3:**

 On page 20, the authors argue that a systematic overview *“can help determine which rebound effects and mitigation strategies fall within the zone of influence of practitioners”. *The revised manuscript expands the discussion of practical implications in Sections 4.1 and 4.2 in response to earlier reviewer feedback, and these improvements are acknowledged. However, the issue raised here is different from the question of whether the practical implications are sufficiently detailed. It concerns a structural gap in the paper’s underlying analytical logic, which remains unresolved in the revised version.

 Table 4 distinguishes between mitigation strategies at the Business and Policy levels, which can be seen as a partial step toward clarifying the ‘zone of influence’. However, the paper does not clearly explain the logic linking the three rebound types — direct, indirect, and economy-wide — to the business/policy distinction. The sentence added in Section 4.1 notes that *“categorizing rebound effects as direct, indirect, or economy-wide helps assess how innovations impact different economic levels and interact”*, but this describes the usefulness of the typology rather than the logic for mapping rebound types to the ‘zone of influence’.

 For example, the paper does not explain why economy-wide rebound effects primarily require policy responses, or how practitioners should determine whether a particular indirect rebound falls within their organization’s ‘zone of influence’. The same gap appears in Section 4.2, where practitioners are advised to “ *identify and address potential rebound effects early in business model design”,* yet no guidance is provided on how the direct/indirect/economy-wide classification should inform that identification process in practice.

 If the authors want the ‘zone of influence’ framing to function as a substantive analytical contribution rather than a rhetorical device, they need to explain explicitly how rebound types connect to the ‘zone of influence’. Even a simple matrix or a short, structured paragraph showing how direct, indirect, and economy-wide rebounds correspond to business and policy domains, and why, would strengthen the argument considerably. Without such clarification, the concept remains more of an assertion than an analytical contribution, and the practical guidance offered to decision makers remains not concrete enough.

**Comment #4:**

 The Introduction explicitly state that *“the focus of this paper is mainly on negative effects*”. In their response to a previous reviewer’s question about positive rebound effects, the authors also clarified that positive rebounds can be socially beneficial and, in some cases, acceptable.

 However, this philosophical acknowledgement does not resolve the methodological inconsistency that remains in the published manuscript. Table 4 includes two entries explicitly labelled as positive rebound effects. Under Product Service Systems, it lists *“greater emotional attachment to second-hand clothes means clothes are discarded less frequently (positive rebound)”,* and under Circular Production & Distribution, it lists *“full battery electric cars and fuel cell electric cars have a higher capital cost, reducing overall consumption (positive rebound)”. *These two items are not discussed in the corresponding results section, nor are they examined analytically in the discussion. More importantly, the methods section does not specify whether positive rebound cases were included or excluded from the systematic analysis, nor does it explain why these two particular cases were retained despite the paper’s stated focus on negative impacts.

 This creates a methodological transparency issue that is separate from the philosophical question of whether positive rebounds matter. The inclusion criteria for RQ1 (p. 5) define the scope as the empirical measurement and identification of rebound effects, but they do not indicate how positive rebound cases should be handled. If positive rebounds are considered within scope, the methods section should state this explicitly and explain the selection criteria. If they are out of scope but retained for contextual purposes, this decision should be clearly noted in the methods or results section rather than left implicit. As currently presented, the two positive rebound entries in Table 4 exist in a methodological grey area, neither fully included within the paper’s declared scope nor formally excluded.

**Comment #5:**

 Section 2.2 does not provide any information about the safeguards used to ensure the reliability of the qualitative coding. The manuscript does not mention whether multiple researchers coded the materials independently, what steps were taken to minimise subjective interpretation bias, or whether any formal measures of inter-coder reliability (e.g. Cohen’s Kappa or percentage agreement) were assessed. The authors should therefore explain how coding disagreements were handled within the research team, outline the procedures used during the coding process, and provide empirical evidence that supports the reliability of the coding.

**Comment #6:**

 On page 20, second paragraph, the sentence reads, *“Indeed, only 32 of 45 papers linked mitigation strategies to specific rebound effects.”* But the methods section (p. 5) states that the mitigation sample consists of 32 papers, not 45. Since the manuscript never explains what the denominator of 45 refers to, it is unclear whether this number represents a combined sample, some other subset, or simply a counting error. This inconsistency needs to be clarified.

Are sufficient details of the methods and analysis provided to allow replication by others?

Partly

Are the rationale for, and objectives of, the Systematic Review clearly stated?

Yes

Is the statistical analysis and its interpretation appropriate?

Yes

Are the conclusions drawn adequately supported by the results presented in the review?

Yes

Reviewer Expertise:

circular business models, green human resource management

I confirm that I have read this submission and believe that I have an appropriate level of expertise to confirm that it is of an acceptable scientific standard, however I have significant reservations, as outlined above.

# Reviewer report: Reviewer response for version 2

**DOI:** 10.21956/openreseurope.24781.r73799

**Published:** 2026-06-10

**Authors:** Mariantonietta Ferrante

**Affiliations:** 1 Politecnico di Bari, Bari, Italy

I appreciate the careful revision and the way my comments were taken into account. I have no further remarks.

Are sufficient details of the methods and analysis provided to allow replication by others?

Yes

Are the rationale for, and objectives of, the Systematic Review clearly stated?

Yes

Is the statistical analysis and its interpretation appropriate?

Yes

Are the conclusions drawn adequately supported by the results presented in the review?

Partly

Reviewer Expertise:

Circular economy, Circular manufacturing, Rebound effects

I confirm that I have read this submission and believe that I have an appropriate level of expertise to confirm that it is of an acceptable scientific standard.

# Reviewer report: Reviewer response for version 2

**DOI:** 10.21956/openreseurope.24781.r74517

**Published:** 2026-06-08

**Authors:** Koen Dittrich

**Affiliations:** 1 Erasmus University, Rotterdam, South Holland, The Netherlands

**1. Contribution and Originality**

 The manuscript addresses a highly relevant and increasingly important issue within circular economy scholarship: the persistence and management of rebound effects in circular business models (CBMs). Its principal contribution lies in systematically organizing empirically identified rebound effects according to specific circular business model archetypes. This constitutes a meaningful advancement over prior reviews, which have generally treated rebound effects at the level of the circular economy broadly, without explicitly linking them to business model configurations.

 The manuscript’s attempt to bridge sustainability transitions literature, rebound economics, and business model innovation is commendable. In particular, the archetype-based classification offers a potentially useful analytical structure for both researchers and practitioners. By mapping specific rebound mechanisms to archetypes such as Product-Service Systems, Next Life models, and Collaborative Consumption, the paper provides an accessible synthesis that could help organizations anticipate rebound vulnerabilities during business model design.

 The practical orientation of the manuscript also enhances its relevance. The emphasis on ex-ante mitigation during business experimentation reflects an important gap in the literature, especially given that many sustainability assessments occur only after implementation. The manuscript appropriately recognizes that design-stage decisions shape long-term environmental outcomes and lock-in effects.

 However, while the contribution is original in structure, it is somewhat limited in analytical ambition. The paper primarily organizes existing knowledge rather than developing a novel explanatory theory of why specific business model archetypes generate particular rebound dynamics. In this sense, the manuscript contributes more strongly as a synthesis and classification exercise than as a theoretical advancement. A stronger conceptual contribution would emerge if the paper moved beyond categorization toward mechanism-based theorization of rebound generation across different circular configurations.

 Additionally, the novelty claim should be articulated more carefully. While the manuscript may indeed be among the first to systematically classify rebounds by CBM archetypes, related streams of work in industrial ecology, sufficiency research, and sustainable consumption studies have explored adjacent relationships between circular strategies and systemic behavioral responses. Positioning the paper more precisely within these debates would strengthen the originality claim.

 Overall, the contribution is substantial and potentially publishable, but its theoretical significance would benefit from deeper analytical synthesis and stronger articulation of the mechanisms connecting circular business model design and rebound emergence.

**2. Theoretical Framing**

 The manuscript demonstrates extensive familiarity with the rebound effect literature and effectively situates itself within current circular economy and sustainability transition debates. The literature review is broad, interdisciplinary, and generally up to date. The authors successfully integrate literature from industrial ecology, product-service systems, sustainable business models, consumer behavior, and circular economy research into a coherent narrative framework.

 One notable strength is the manuscript’s recognition that rebound effects are systemic phenomena rather than isolated behavioral anomalies. The discussion appropriately emphasizes that circular strategies can unintentionally increase environmental impact through mechanisms such as efficiency gains, cost reductions, imperfect substitution, moral licensing, and induced demand. This systems-oriented framing aligns well with contemporary rebound scholarship.

 The decision to use the Pieroni et al. archetypal framework is also defensible. The authors justify the selection on the basis that the framework differentiates upstream and downstream circular strategies more clearly than competing typologies. This rationale is methodologically sound and enhances analytical consistency across the review.

 Nevertheless, the theoretical framing remains conceptually underdeveloped in several important respects.

 The most significant issue concerns conceptual ambiguity surrounding the definition of rebound effects themselves. Throughout the manuscript, the boundaries between rebound effects, unintended consequences, spillover effects, induced demand, moral licensing, substitution failures, and broader systemic externalities remain unclear. While the authors acknowledge this problem in the Discussion section, the ambiguity is not resolved analytically.

 This conceptual instability weakens the rigor of the synthesis because several phenomena categorized as rebound effects may not satisfy stricter definitions commonly used in rebound economics literature. Some examples represent classical rebound mechanisms tied to efficiency gains and price elasticity, while others reflect broader behavioral or institutional responses not directly reducible to rebound dynamics. The review therefore risks conceptual overextension.

 The manuscript would benefit substantially from a more formal conceptual architecture introduced early in the paper. Specifically, the authors should explicitly distinguish:

- direct rebound effects,
- indirect rebound effects,
- economy-wide rebound effects,
- behavioral spillovers,
- moral licensing,
- induced consumption,
- substitution failures,
- and unintended consequences.

At present, these categories overlap too heavily, creating analytical imprecision.

 Another limitation is the relative absence of deeper theoretical mechanism-building. The manuscript catalogs numerous rebound phenomena but does not sufficiently explain why certain archetypes appear particularly vulnerable to specific rebound mechanisms. For instance, Product-Service Systems are repeatedly associated with careless consumption and intensified usage, yet the paper stops short of theorizing the structural features of ownership transfer, incentive realignment, or psychological detachment that generate these outcomes. Similarly, the review identifies efficiency-induced demand increases across multiple archetypes but does not develop a broader explanatory model connecting circular efficiency logics to consumption expansion.

 The manuscript also engages only minimally with critical perspectives on circular economy discourse. There is limited engagement with degrowth literature, sufficiency theory, political economy critiques of green growth, or deeper debates surrounding Jevons paradox. Incorporating these perspectives would substantially enrich the theoretical framing and position the paper more centrally within broader sustainability transition debates.

 In summary, the theoretical framing is broad and competent but remains primarily descriptive rather than analytically generative. Greater conceptual precision and deeper mechanism-based theorization would significantly improve the manuscript’s scholarly rigor.

**3. Research Questions**

 The research questions are clearly stated and logically aligned with the structure of the review. The first question—concerning the potential rebound effects of different circular business model archetypes—is particularly strong and appropriately scoped for a systematic review approach. It provides a clear organizing principle for the empirical synthesis.

 The second research question, concerning mitigation strategies, is also relevant and practically important. However, its formulation is somewhat limited because it encourages descriptive cataloguing rather than analytical evaluation. By asking merely “what strategies have been identified,” the paper implicitly treats all proposed mitigation strategies as epistemically equivalent, regardless of evidentiary strength.

 This becomes problematic later in the manuscript, where empirically observed rebound effects are juxtaposed with largely untested mitigation proposals. The asymmetry between empirical rigor on the rebound side and speculative suggestion on the mitigation side weakens the coherence of the review.

 A stronger formulation of the second research question might have emphasized:

- mechanisms of mitigation,
- contextual effectiveness,
- empirical validation,
- or conditions under which mitigation strategies succeed or fail.

Such reframing would have pushed the analysis toward a more evaluative synthesis rather than a broad inventory of suggestions.

 Nonetheless, the research questions are generally coherent and appropriate for the study’s objectives.

**4. Methodological Rigor**

 The methodological section demonstrates several strengths. The authors provide a reasonably transparent overview of the search process, including search strings, databases, screening stages, snowballing procedures, and coding categories. The use of a PRISMA-style flow diagram improves transparency and aligns the review with accepted systematic review conventions.

 The coding framework is also relatively comprehensive. Including dimensions such as sector, geographical scope, methodology, rebound type, archetype classification, and mitigation strategies creates a robust descriptive dataset capable of supporting meaningful synthesis.

 However, despite these strengths, the methodological rigor remains one of the manuscript’s most significant weaknesses.

 The paper labels itself a “systematic literature review,” yet methodologically it operates closer to a semi-systematic or structured qualitative synthesis. Several elements commonly expected in rigorous systematic reviews are either absent or insufficiently operationalized.

 Most notably, the manuscript does not report any formal inter-coder reliability assessment. Although the authors mention that coding was periodically checked by co-authors, there is no reporting of:

- Cohen’s kappa,
- Krippendorff’s alpha,
- percentage agreement,
- or reconciliation procedures.

Given the interpretive complexity involved in classifying rebound mechanisms and assigning archetypes, this omission significantly weakens the reliability of the coding process.

 The inclusion and exclusion criteria also remain under-specified. The requirement that studies demonstrate “clear empirical measurement” is insufficiently operationalized. It is unclear:

- what qualifies as empirical evidence,
- whether simulation studies are treated equivalently to observational studies,
- how scenario analyses were evaluated,
- or how conceptual extrapolations were excluded.

This ambiguity raises concerns about consistency in article selection.

 Additionally, the search strategy itself presents recall and precision concerns. The search strings are extremely broad, particularly the inclusion of terms such as “business*,” “indirect effect*,” and “secondary effect*.” Such breadth likely generated substantial irrelevant noise and may have increased subjectivity during screening.

 The review also lacks a formal quality appraisal framework for included studies. No attempt is made to distinguish stronger empirical evidence from weaker or more speculative studies. As a result, highly heterogeneous evidence types—including conceptual papers, LCAs, simulation models, surveys, and case studies—are synthesized without methodological weighting. This undermines the robustness of the conclusions.

 Finally, there is an important epistemological asymmetry between RQ1 and RQ2. While rebound effects are restricted to empirically measured phenomena, mitigation strategies are often drawn from conceptual or normative recommendations without empirical validation. This asymmetry should be acknowledged more explicitly and analytically addressed.

 Overall, while the methodology is transparent and systematic in spirit, it falls short of the rigor typically expected from high-quality systematic reviews in peer-reviewed journals.

**5. Data Analysis and Synthesis**

 The manuscript’s synthesis work is ambitious and clearly represents a substantial effort. The archetype-based organization of rebound effects and mitigation strategies is intuitive, accessible, and potentially useful for future scholarship and practical application. Table 4, in particular, serves as the manuscript’s central integrative contribution.

 The categorization of rebound effects into direct, indirect, and economy-wide forms also adds analytical structure and helps clarify the different scales at which rebound mechanisms operate.

 However, the synthesis remains largely descriptive rather than analytical. Much of the Results section consists of:

- cataloguing examples,
- summarizing findings,
- and aggregating mitigation proposals.

The manuscript rarely moves toward higher-order analytical interpretation. For example, while recurring patterns are observable across archetypes—especially efficiency-induced demand growth and price-mediated consumption expansion—the paper does not sufficiently theorize these recurring dynamics.

 Similarly, there is little comparative analysis between archetypes. Why are Product-Service Systems especially vulnerable to careless consumption? Why do Next Life strategies repeatedly produce moral licensing effects? Why do efficiency-oriented archetypes generate economy-wide rebounds more frequently? These questions are implied but not systematically analyzed.

 Another important weakness concerns evidentiary weighting. All findings appear to be treated equally regardless of methodological robustness. A single case study and a large-scale quantitative assessment receive comparable narrative emphasis. Without quality differentiation, the synthesis risks overstating the certainty and generalizability of some findings.

 Table 4 exemplifies both the strengths and weaknesses of the manuscript. It is impressive in scope and likely to attract attention from readers and practitioners. At the same time, it conflates:

- empirically demonstrated rebound effects,
- speculative mitigation ideas,
- conceptual recommendations,
- and normative policy suggestions.

Some mitigation proposals appear weakly substantiated or conceptually vague. Examples such as “design products that feel expensive” or “use rewards and fines” require stronger theoretical grounding and contextual explanation.

 The manuscript would benefit from:

- distinguishing empirically validated mitigation strategies from speculative proposals,
- clustering mechanisms more systematically,
- and explicitly discussing evidence strength.

As it stands, the synthesis is highly informative but analytically underdeveloped.

**6. Use of Sources and Citations**

 The manuscript demonstrates broad engagement with relevant literature and includes a substantial number of contemporary references. The citation base spans multiple disciplines and reflects awareness of recent developments in circular economy and rebound effect scholarship.

 The integration of industrial ecology, business model innovation, sustainability transitions, and behavioral research is particularly commendable. The manuscript also incorporates several recent publications from 2024–2026, suggesting strong engagement with emerging debates.

 However, the literature integration occasionally becomes overly accumulative. Many sections follow a repetitive structure in which individual studies are sequentially summarized without sufficient synthesis or comparison. As a result, citation density sometimes substitutes for analytical depth.

 The manuscript would benefit from more explicit engagement with:

- contradictions between studies,
- methodological differences,
- contextual dependencies,
- and competing interpretations.

Additionally, several foundational rebound economics scholars receive relatively limited treatment compared to more recent circular economy literature. Greater engagement with classical rebound scholarship would strengthen theoretical grounding and historical continuity.

 Despite these limitations, the literature review remains one of the stronger aspects of the manuscript overall.

**7. Discussion Section**

 The Discussion section effectively reiterates the practical significance of rebound effects in circular business models and appropriately emphasizes the complexity of sustainability transitions. The recognition that rebound effects may be inherent to circular economy strategies is an important and intellectually honest conclusion.

 The manuscript also deserves credit for acknowledging social justice implications. The discussion surrounding carbon taxes, price controls, and the disproportionate burden placed on lower-income groups introduces a valuable ethical dimension often absent in circular economy research.

 Similarly, the acknowledgment that some rebound effects may be socially acceptable—or even desirable—in contexts such as alleviating energy poverty demonstrates commendable nuance.

 Nevertheless, the Discussion section remains relatively limited in theoretical ambition. Much of the section restates empirical observations rather than elevating them into broader conceptual insights.

 The paper would benefit substantially from:

- a clearer conceptual model,
- stronger mechanism theorization,
- or a framework linking archetype characteristics to rebound susceptibility.

For example, the authors could potentially develop propositions concerning:

- ownership structures,
- pricing mechanisms,
- perceived abundance,
- substitutability,
- or user attachment as drivers of rebound vulnerability.

Without such theoretical elevation, the Discussion remains primarily reflective rather than generative.

**8. Writing Quality**

 The manuscript is generally well written and structurally coherent. The organization follows a logical progression from background to methodology, results, discussion, and conclusion. The writing is accessible despite the conceptual complexity of the topic.

 However, the manuscript would benefit from significant stylistic tightening. Several sections are repetitive, particularly around:

- efficiency gains,
- consumer awareness,
- price effects,
- and overconsumption.

The paper could likely be reduced by approximately 15–20% without loss of substantive content.

 There are also occasional grammatical and formatting inconsistencies, including typographical errors, inconsistent capitalization, awkward sentence constructions, and uneven table formatting. While these are not severe, they collectively reduce perceived polish and professionalism.

 Table 4, although highly valuable, is extremely dense and difficult to navigate. It may need restructuring, visual simplification, or partial relocation to supplementary materials.

 Overall, the writing quality is solid but would benefit from careful editorial refinement.

**9. Overall Evaluation**

 Overall, the manuscript is ambitious, timely, and potentially impactful. Its greatest strength lies in its systematic organization of rebound effects and mitigation strategies across circular business model archetypes. The paper fills a meaningful gap in the literature and demonstrates broad interdisciplinary engagement.

 However, the manuscript currently functions more effectively as a structured mapping review than as a deeply analytical systematic review. The central weaknesses concern conceptual ambiguity, methodological limitations, insufficient evidentiary differentiation, and limited mechanism-based theorization.

 The paper has strong potential, but realizing that potential will require substantial revision focused on:

- conceptual clarification,
- analytical deepening,
- methodological strengthening,
- and theoretical integration.

In its current form, I would give the manuscript a “major revisions” decision before being ready for indexing on an academic open science platform.

Are sufficient details of the methods and analysis provided to allow replication by others?

Yes

Are the rationale for, and objectives of, the Systematic Review clearly stated?

Yes

Is the statistical analysis and its interpretation appropriate?

Partly

Are the conclusions drawn adequately supported by the results presented in the review?

Partly

Reviewer Expertise:

circular economy, sustainable business models, closed-loop supply chains

I confirm that I have read this submission and believe that I have an appropriate level of expertise to confirm that it is of an acceptable scientific standard, however I have significant reservations, as outlined above.

# Reviewer report: Reviewer response for version 2

**DOI:** 10.21956/openreseurope.24781.r73798

**Published:** 2026-06-08

**Authors:** Vivian S.C Tunn

**Affiliations:** 1 Utrecht University, Utrecht, The Netherlands

First of all, I would like to thank you for the thorough, thoughtful revision and the clear explanations of the changes in the response to reviewers. The manuscript has significantly improved through the revision. Thank you for this interesting and well-written contribution.

 From my side, the manuscript is approved and ready for indexing

Are sufficient details of the methods and analysis provided to allow replication by others?

Yes

Are the rationale for, and objectives of, the Systematic Review clearly stated?

Yes

Is the statistical analysis and its interpretation appropriate?

Yes

Are the conclusions drawn adequately supported by the results presented in the review?

Yes

Reviewer Expertise:

NA

I confirm that I have read this submission and believe that I have an appropriate level of expertise to confirm that it is of an acceptable scientific standard.

# Reviewer report: Reviewer response for version 2

**DOI:** 10.21956/openreseurope.24781.r73795

**Published:** 2026-06-03

**Authors:** Christian Koldewey

**Affiliations:** 1 Paderborn University, Paderborn, Germany

Dear Authors,

 Thank you for the thorough revision of your manuscript. Although the search string itself was not further optimized, its current form has now been sufficiently justified and explained.

 In our view, the manuscript has reached a very good state. We therefore fully support its approval.

Are sufficient details of the methods and analysis provided to allow replication by others?

Partly

Are the rationale for, and objectives of, the Systematic Review clearly stated?

Yes

Is the statistical analysis and its interpretation appropriate?

Yes

Are the conclusions drawn adequately supported by the results presented in the review?

Yes

Reviewer Expertise:

Circular Business Models, Circular Product Design

I confirm that I have read this submission and believe that I have an appropriate level of expertise to confirm that it is of an acceptable scientific standard.

# Reviewer report: Reviewer response for version 2

**DOI:** 10.21956/openreseurope.24781.r73796

**Published:** 2026-06-02

**Authors:** Joël Ntsondé

**Affiliations:** 1 ISTEC Business School, Paris, France

Thank you for thoroughly addressing my questions and for taking my comments into account. I have no further remarks.

Are sufficient details of the methods and analysis provided to allow replication by others?

Yes

Are the rationale for, and objectives of, the Systematic Review clearly stated?

Yes

Is the statistical analysis and its interpretation appropriate?

Partly

Are the conclusions drawn adequately supported by the results presented in the review?

Partly

Reviewer Expertise:

Circular business models, sustainable & responsable innovation, transition studies

I confirm that I have read this submission and believe that I have an appropriate level of expertise to confirm that it is of an acceptable scientific standard.

# Reviewer report: Reviewer response for version 2

**DOI:** 10.21956/openreseurope.24781.r73797

**Published:** 2026-06-02

**Authors:** Konstantin Remke

**Affiliations:** 1 Strategy & Entrepreneurship, Ecole de Management de Normandie, Clichy, Paris, Normandy, France; 2 ESCP Business School, Paris, France

**Summary and General Remarks**

 Thank you for the opportunity to review the revised manuscript "Rebound effects in circular business models: A review of cases and mitigation strategies."

 The manuscript reviews extant literature on circular economy rebound effects, identifies direct and indirect effects across different circular business model archetypes, and extracts mitigation strategies documented in the literature. The topic is timely and the overall research design remains appropriate for the study's objectives.

 Compared to the previous submission, the authors have made a number of notable improvements. Most importantly, they have removed the design science framing from the manuscript (a claim that was previously difficult to substantiate given the study's methodological nature) and have integrated Mueller & Remke (2025), which had been a significant omission in the first round. These revisions are appreciated. However, several concerns from the first round remain inadequately addressed, and new concerns have emerged from this revision. The most pressing of these relates to the methodological rigor of the literature review, the underdeveloped discussion section, and the overall weakness of the manuscript's stated contributions. These issues must be addressed before the manuscript can be considered for publication.

**Major Comments**

**1. Methodological Grounding of the Literature Review**

 The authors rely on Bryman and Bell (2011) as the methodological foundation for their systematic literature review. While this is a widely cited reference, it is a non-peer-reviewed textbook that addresses a broad range of business research methods and does not constitute an appropriate methodological anchor for a systematic literature review. The authors are strongly encouraged to instead draw from Tranfield et al. (2003), which provides a well-established and domain-appropriate protocol for systematic literature reviews in management and organization research, or alternatively from Post et al. (2020), which offers a more recent methodological framework suited to this type of study. Grounding the review methodology in one of these references would substantially improve the credibility and rigor of the study design.

**2. Transparency of the Search Strategy and Temporal Scope**

 The rationale for limiting the search to articles indexed on Web of Science and Scopus until September 2024 is not adequately explained. While both databases are appropriate choices, the authors should articulate why September 2024 was selected as the cutoff and whether this decision was theoretically or practically motivated. Moreover, the article and journal selection procedures remain insufficiently described. This is a critical issue: without clear inclusion and exclusion criteria (including how journals were evaluated for relevance and quality) the credibility of the resulting sample, and by extension the study's findings, is weakened. The previous review raised concern that many reviewed papers appear in lower-tier journals; this issue has not yet been addressed. The authors should describe their journal selection criteria explicitly and, where appropriate, consider whether quality thresholds (e.g., journal rankings or citation indices) were applied. The authors should also clarify what is meant by "articles of non-relevant research areas" as an exclusion criterion and provide at least one illustrative example to guide the reader.

**3. Insufficient Explanation of the Article Screening Funnel**

 The drop from 1,091 articles to 74, and subsequently to 32 articles included in the final sample, represents a significant reduction that is not adequately explained. The screening process and the criteria applied at each stage should be described transparently and in sufficient detail to allow the reader to assess the plausibility of the selection decisions. Additionally, the authors are encouraged to restate the research questions (RQs) within the method section itself. In their current form, the review steps are described without a clear and explicit connection to the RQs, which makes it difficult for the reader to evaluate whether the review procedure is aligned with the study's stated objectives.

**4. Structural Issue in Section 3**

 Section 3.2, "Rebound effects of circular business archetypes and associated potential mitigation strategies," currently functions as the overarching header for Sections 3.3 through 3.10. This creates a structural inconsistency, as Sections 3.3–3.10 are positioned as parallel to 3.2 rather than as subsections of it. The appropriate correction is to restructure Sections 3.3–3.10 as Sections 3.2.1–3.2.8, positioned as subordinate to the overarching Section 3.2. Additionally, Section 3 should be renamed from "Results" to "Findings," as the term "Results" is conventionally reserved for quantitative studies, while "Findings" is more appropriate for qualitative and review-based research of this nature.

**5. Characterization of the Circular Systems Sandbox**

 The authors' characterization of the Circular Systems Sandbox (Mueller & Remke, 2025) as not archetype-specific is somewhat misleading. While it is accurate that the tool focuses on the process of business model design rather than on archetype-specific rebound effects, it nonetheless explicitly connects business model choices (including references to the circular business model archetypes outlined by Pieroni et al. (2020)) to rebound effects, and its efficacy was tested in a field experiment. The manuscript's current framing risks misrepresenting this artifact's scope and empirical grounding. The authors should revise the characterization accordingly to more accurately reflect what the tool does and does not address.

**6. Discussion Quality and Contribution Clarity**

 This remains the most critical concern with the manuscript. The discussion section continues to function largely as a repetition of the findings rather than as a space for theoretical synthesis and contribution. The authors should restructure the discussion to clearly distinguish between: (a) what the study found, (b) how the findings advance or extend extant theoretical conversations on circular economy rebound effects and circular business model design, and (c) what the practical implications are for managers and policymakers. The theoretical contribution described in Section 4.2 is a step in the right direction, but it remains underdeveloped and does not yet connect convincingly with the broader literature on circular economy rebound mitigation. In the current form, the manuscript's contributions do not justify the scope of the review. The authors should take care to clearly articulate what the study adds to the conversation that was not already known, and substantiate these claims with specific reference to the study's findings.

**Minor Comments**

- In the introduction, circular business model literature consistently frames these models in terms of slowing, narrowing, or closing resource loops (Bocken et al., 2016). Within this framework, regeneration is more appropriately categorized under the closing of resource loops rather than as a separate category. This framing should be corrected in the introduction to align with established terminology in the field.
- The manuscript contains recurring grammatical errors, most commonly missing commas in complex sentences. For example: "Past research has found that negative side effects of circular business models can potentially be prevented during the design and experimentation phase so understanding these is essential (Das et al., 2023)" — a comma is required before "so." A thorough proofreading pass is recommended prior to resubmission.

I hope these comments help the authors to further improve the manuscript.

Are sufficient details of the methods and analysis provided to allow replication by others?

Partly

Are the rationale for, and objectives of, the Systematic Review clearly stated?

Yes

Is the statistical analysis and its interpretation appropriate?

Partly

Are the conclusions drawn adequately supported by the results presented in the review?

Partly

Reviewer Expertise:

Circular economy, circular business model innovation, circular economy rebound mitigation, circular systems design, design science research

I confirm that I have read this submission and believe that I have an appropriate level of expertise to confirm that it is of an acceptable scientific standard, however I have significant reservations, as outlined above.

## References

[1] : Advancing Theory with Review Articles. *Journal of Management Studies* .2020;57(2) : 10.1111/joms.12549 351-376 10.1111/joms.12549

[2] : Towards a Methodology for Developing Evidence‐Informed Management Knowledge by Means of Systematic Review. *British Journal of Management* .2003;14(3) : 10.1111/1467-8551.00375 207-222 10.1111/1467-8551.00375

[3] : Circular Systems Sandbox: A process tool for circular systems design and rebound effect reduction. *Journal of Business Venturing Design* .2025;4: 10.1016/j.jbvd.2025.100028 10.1016/j.jbvd.2025.100028

# Reviewer report: Reviewer response for version 2

**DOI:** 10.21956/openreseurope.24781.r73945

**Published:** 2026-05-13

**Authors:** Teresa Paiva

**Affiliations:** 1 BRIDGES - Biotechnology Research, Innovation and Design for Health Products, Instituto Politecnico da Guarda, Guarda, Guarda District, Portugal

This manuscript addresses an increasingly relevant topic within circular economy and sustainability research by systematically reviewing rebound effects associated with circular business model archetypes and the mitigation strategies proposed in the literature. The paper offers a valuable synthesis of empirical and conceptual research, helping bridge the gap between circular business model innovation and rebound effect mitigation.

 The manuscript is timely, relevant, and generally well structured. Its principal contribution lies in organising rebound effects according to specific circular business model archetypes and consolidating mitigation strategies from both business and policy perspectives. The topic is highly pertinent for researchers, practitioners, and policymakers engaged in circular economy transitions.

 The introduction successfully contextualises the growing relevance of rebound effects in circular economy research and establishes the importance of understanding unintended consequences within circular business model innovation. The manuscript effectively highlights the tension between sustainability intentions and actual environmental outcomes.

 The discussion of circular business model archetypes and rebound mechanisms is generally relevant and well-connected to recent literature. The integration of the work of Pieroni et al. (2020), Zink & Geyer (2017), and more recent rebound-effect scholarship strengthens the study's positioning.

 However, several aspects could be improved:

- The theoretical discussion of rebound effects remains somewhat descriptive and could benefit from deeper conceptual clarification regarding: direct, indirect, economy-wide, and systemic rebound effects.
- Although the revised manuscript introduces a clearer definition of rebound effects, the distinction between rebound effects, unintended consequences, and broader systemic externalities still requires greater analytical precision.
- The introduction would benefit from a more detailed explanation of the seven circular business model archetypes earlier in the paper, as they become central to the analytical framework.
- The manuscript relies heavily on literature connected to the authors' prior work. While understandable given their expertise in the field, broader engagement with adjacent sustainability transition and consumption literature would strengthen the theoretical positioning.
- The contribution relative to previous reviews, particularly Baczyk et al. (2024), should continue to be clarified carefully to avoid overstating novelty.

Overall, the introduction is strong and relevant, but the conceptual framing would benefit from greater theoretical depth and sharper distinctions between key concepts.

 The systematic review methodology is generally appropriate and clearly aligned with the study objectives. The use of Scopus and Web of Science, combined with structured search strings and coding procedures, provides a solid basis for the review.

 The inclusion of coding categories such as sector, rebound magnitude, business model archetype, and mitigation strategies is particularly useful and contributes to the paper's analytical structure.

 Nevertheless, several methodological aspects require clarification:

1. The manuscript would benefit from a more explicit explanation of: inclusion and exclusion criteria, screening decisions, and how disagreements between authors were resolved.
2. Greater detail on coding consistency and validation procedures would strengthen confidence in the analytical reliability.
3. Although the search strategy is transparent, relying on only two databases may have constrained the comprehensiveness of the review, as acknowledged by the authors.
4. The manuscript would benefit from additional explanation regarding how mitigation strategies were synthesised and categorised into business- versus policy-oriented approaches.
5. The distinction between empirical evidence and conceptual propositions should be more consistently maintained throughout the analysis.

Overall, the methodology is appropriate and reasonably transparent, though further detail would improve reproducibility and analytical rigour.

 Circular business model archetypes clearly organise the results and provide a useful overview of the rebound mechanisms associated with each category.

 The categorisation of rebound effects into direct, indirect, and economy-wide is analytically valuable and facilitates interpretation.

 The discussion of mitigation strategies is also relevant and practically useful, especially for practitioners engaged in circular business model experimentation.

 However, some improvements are recommended:

- Several sections remain highly descriptive and would benefit from deeper comparative synthesis across archetypes.
- Some archetypes, particularly "long life" and "next life," appear conceptually overlapping and should be more clearly differentiated.
- More concrete examples summarising how specific rebound effects emerge within particular business models would improve readability.
- The manuscript would benefit from a synthesis table explicitly linking archetypes, rebound mechanisms, mitigation strategies, and evidence strength.
- Greater differentiation between empirically observed mitigation strategies and speculative recommendations would improve interpretive clarity.

The results section is comprehensive and informative, but could be more analytically integrated.

 The discussion appropriately emphasises that rebound effects may be an inherent feature of circular business models and highlights the importance of integrating awareness of rebound effects into business model design.

 The manuscript also makes an important contribution by discussing the limitations of current mitigation approaches and the risks of generalised policy interventions.

 Particularly valuable is the recognition that mitigation strategies may themselves trigger additional rebounds, and that social justice implications must be considered when designing restrictive consumption policies.

 Nevertheless, the discussion could be strengthened further by:

- providing more critical reflection on the tension between sufficiency-oriented approaches and market-based growth logics;
- engaging more explicitly with sustainability transitions and socio-technical systems perspectives;
- discussing the practical feasibility of implementing proposed mitigation strategies in different regulatory and market contexts;
- and clarifying how practitioners can operationalise the findings during business model experimentation.

The discussion is thoughtful and relevant, but deeper critical engagement would strengthen the paper's theoretical contribution.

 The conclusion is coherent, balanced, and adequately supported by the findings. The manuscript appropriately avoids overstating the effectiveness of mitigation strategies and clearly acknowledges the lack of empirical validation.

 The limitations section is transparent and well-developed, particularly regarding database limitations, contextual specificity, and uncertainty about mitigation effectiveness.

 However, additional limitations could be acknowledged, including:

- publication bias toward well-established circular business models,
- the predominance of European and North American case studies,
- and the limited integration of Global South perspectives.

The conclusion could also provide a stronger forward-looking research agenda regarding:

- standardised rebound assessment methods,
- integration into business model experimentation,
- and policy evaluation frameworks.

To sum up, there are some recommendations for improvement:

1. Strengthen conceptual distinctions between rebound effects, unintended consequences, and systemic externalities.
2. Provide clearer definitions and differentiation of circular business model archetypes.
3. Expand the theoretical framing beyond descriptive literature synthesis.
4. Clarify coding reliability and article selection procedures.
5. Improve analytical synthesis across archetypes rather than presenting findings mainly descriptively.
6. Differentiate more clearly between empirically tested and speculative mitigation strategies.
7. Add synthesis tables connecting archetypes, rebound effects, and mitigation strategies.
8. Expand discussion of socio-technical and sustainability transition perspectives.
9. Further clarify the manuscript's novelty relative to prior reviews.
10. Include a stronger discussion of contextual and geographical limitations.

Are sufficient details of the methods and analysis provided to allow replication by others?

Partly

Are the rationale for, and objectives of, the Systematic Review clearly stated?

Yes

Is the statistical analysis and its interpretation appropriate?

Yes

Are the conclusions drawn adequately supported by the results presented in the review?

Yes

Reviewer Expertise:

green marketing and circular economy marketing; innovation management and entrepreneurship

I confirm that I have read this submission and believe that I have an appropriate level of expertise to confirm that it is of an acceptable scientific standard, however I have significant reservations, as outlined above.

# Reviewer report: Reviewer response for version 1

**DOI:** 10.21956/openreseurope.23046.r62339

**Published:** 2025-11-10

**Authors:** Joël Ntsondé

**Affiliations:** 1 ISTEC Business School, Paris, France

Based on a systematic literature review of 76 articles, the objective of the paper is to connect the literature on rebound effects in circular business models (CBMs) with that on circular business model archetypes and design strategies aimed at limiting these effects—whether direct, indirect, or economy-wide.

 I thank the editors for inviting me to review this paper and the authors for undertaking research on such a fundamental topic.

 The paper is very interesting, clear, and important for advancing the literature on circular business models and the issue of rebound effects.

 The literature review and methodology are well structured and rigorous.

 The literature gap is clearly identified, as is the paper’s potential contribution.

 My main comments concern the Results and Discussion sections, where certain points would benefit from further clarification or elaboration.

 I hope these comments will help the authors deepen their research and advance the literature on rebound effects, a crucial topic for the ecological transition.

**Methodology**

 Figures 2 and 3: It would be useful to discuss potential biases related to the geographical and sectoral distribution of the selected articles, in order to assess their possible impact on the results.

 The category “unspecified” seems to include literature reviews or conceptual papers, whereas the main selection criterion for the articles is the empirical identification of rebound effects. This point is not entirely clear: it would be useful to specify how these papers were considered in the analysis.

**Results**

 The classification presented in Table 4 deserves to be revisited:

- The “measures to reduce impact of consumption” do not all seem relevant for discussing strategies to reduce rebound effects, as they do not directly act on economic behavior. It would be preferable to rename them, for instance, “rebound compensation strategies.”
- In the “Other measures” category, some measures should be better justified by explaining how they actually reduce rebound effects (see detailed comments below).
- Rebound effects are particularly evident for “Next life” business models, probably because they are more widespread and better documented.
  For other archetypes, the effects and associated strategies are less explicit and could be described in more detail.

**Discussion**

 The discussion provides a good synthesis of the results and contributions to the literature.

 However, rather than merely summarizing the findings, it would be useful to adopt a more critical analysis of the relevance of the proposed mitigation strategies.

 The paper rightly notes that some studies adopt an overly broad understanding of rebound effects, but it would be valuable to go further by discussing the validity and feasibility of the identified mitigation strategies.

 Some measures mainly target high-income consumers (e.g., *“Design products that feel expensive”*, *“Price control: encourage consumers to pay more for resource-efficient solutions”*). These strategies raise issues of social equity and could even reinforce perceptions of injustice. It would be interesting to build on the already cited works ( *Mies & Gold, 2021; Schröder et al., 2019*) to deepen this discussion.

**Detailed Comments**

**Page 9**

- *“Conduct holistic assessment of the impacts to account for rebound effects”*: Can assessment measures really be considered reduction strategies? This seems debatable.

**Page 10**

- *“Make efforts to change the lifestyles of consumers towards less impactful consumption”*: please specify how this can be implemented.
- *“Use rewards and fines”*: elaborate (what mechanisms, which responsible actors?).
- *“Prevent careless consumption”* and *“Inform consumers about the importance of product care”*: how can these levers be concretely activated?
- *“Integrate multiple CE strategies within a business model”*: why would this help reduce rebound effects?
- *“Eco-efficient value creation”*: should be further detailed.
- *“Design to reduce the need for care”*: wording is unclear.
- *“Introduction of a repairability index for products that can indicate a product’s durability”*: what is the link with rebound effects?
- *“Introduce emission trading rules”* and *“Cap and trade schemes that place an upper limit on environmental stress”*: clarify what these refer to and at what level of action they apply (corporate, public policy, etc.).

**Page 12**

- *“Prevent excessive consumption through sufficiency strategies”*: please specify (what concrete strategies?).

Are sufficient details of the methods and analysis provided to allow replication by others?

Yes

Are the rationale for, and objectives of, the Systematic Review clearly stated?

Yes

Is the statistical analysis and its interpretation appropriate?

Partly

Are the conclusions drawn adequately supported by the results presented in the review?

Partly

Reviewer Expertise:

Circular business models, sustainable & responsable innovation, transition studies

I confirm that I have read this submission and believe that I have an appropriate level of expertise to confirm that it is of an acceptable scientific standard, however I have significant reservations, as outlined above.

# Response

**Published:** 2026-04-22

**Authors:** Ankita Das

**Affiliations:** Not Applicable, The Netherlands

Dear Reviewers,  Thank you for taking the time to review our article and provide your valuable feedback. We believe they have greatly improved the quality of the article. Please see our detailed responses below.

 Kind regards,

 The Authors

**Comment 5.1: **Based on a systematic literature review of 76 articles, the objective of the paper is to connect the literature on rebound effects in circular business models (CBMs) with that on circular business model archetypes and design strategies aimed at limiting these effects—whether direct, indirect, or economy-wide. I thank the editors for inviting me to review this paper and the authors for undertaking research on such a fundamental topic. The paper is very interesting, clear, and important for advancing the literature on circular business models and the issue of rebound effects. The literature review and methodology are well structured and rigorous. The literature gap is clearly identified, as is the paper’s potential contribution. My main comments concern the Results and Discussion sections, where certain points would benefit from further clarification or elaboration. I hope these comments will help the authors deepen their research and advance the literature on rebound effects, a crucial topic for the ecological transition. Methodology Figures 2 and 3: It would be useful to discuss potential biases related to the geographical and sectoral distribution of the selected articles, in order to assess their possible impact on the results. The category “unspecified” seems to include literature reviews or conceptual papers, whereas the main selection criterion for the articles is the empirical identification of rebound effects. This point is not entirely clear: it would be useful to specify how these papers were considered in the analysis.

**Response 5.1: **Thank you for taking the time to review our article, and for your helpful comments. We made the following addition to the limitations section: “It's worth noting that most of the business case study examples found in the literature were from Europe or North American, limiting generalisability of results. As noted by Baczyk et al. (2024), consumer behaviour is often specific to a certain place and culture and it's therefore important to always encourage the development of circular strategies that are tailored to the context within which the business is embedded.” Regarding the unspecified category, these publications were only part of the mitigation sample. For this sample the inclusion criteria was that the publications discussed or researched circular rebound effect mitigation strategies. Since research on mitigation strategies is scarce it seemed less important that these were strictly empirical and more important to include as many sources as possible (as mentioned in Table 2). Therefore publications that lacked empirical identification of rebound effects were not excluded from this sample. We have added the following sentence in Section 3.1 to clarify this further: “The “Unspecified” category encompasses papers that conducted literature reviews, or that were not empirical in nature, e.g. conceptual papers. This primarily included papers in the rebounds mitigation sample, as mentioned in Table 2. ”

**Comment 5.2: **Results The classification presented in Table 4 deserves to be revisited:

- The “measures to reduce impact of consumption” do not all seem relevant for discussing strategies to reduce rebound effects, as they do not directly act on economic behavior. It would be preferable to rename them, for instance, “rebound compensation strategies.”
- In the “Other measures” category, some measures should be better justified by explaining how they actually reduce rebound effects (see detailed comments below).
- Rebound effects are particularly evident for “Next life” business models, probably because they are more widespread and better documented.
  For other archetypes, the effects and associated strategies are less explicit and could be described in more detail.

**Response 5.2: **Thank you for your suggestions. Regarding point 1, these are measures that do not address rebound effects directly, but rather minimise the impact of the rebound once it happens. We have made the following clarification in Section 2.2:  “The suggestions for mitigation were then classified into mitigation strategies for business and policy, and further subdivided into  ‘measures to reduce consumption’, ‘measures to reduce impact of consumption’ (once the rebound effect occurs), and ‘other measures’. ” Regarding point 2, we simply report the mitigation suggestions suggested by articles in our sample. We discuss this in detail in replies to the detailed comments below. Regarding point 3, thank you for this observation. We made the following addition to future research in order to highlight this issue: “Second, the non-identification of rebound effects in a given archetype does not mean they do not exist. Some circular business models are more prevalent and researched than others, for example, the ‘Circular Production & Distribution’ and ‘Next Life’ archetypes had more research studies compared to other archetypes. More research should be directed towards understanding rebound effects in the less well researched circular business archetypes.”

**Comment 5.3:** Discussion The discussion provides a good synthesis of the results and contributions to the literature. However, rather than merely summarizing the findings, it would be useful to adopt a more critical analysis of the relevance of the proposed mitigation strategies. The paper rightly notes that some studies adopt an overly broad understanding of rebound effects, but it would be valuable to go further by discussing the validity and feasibility of the identified mitigation strategies.

**Response 5.3:** Thank you for your comments! Perhaps you are right. Critical analysis is important, of course, however it doesn’t necessarily have a lot to contribute here. The main finding with regard to mitigation strategies is that it is unknown which strategies (or combination of strategies) would actually work. Given that we have a problem without a clear solution, we would argue that all ideas are welcome, at least until further research indicates what may or may not work. Even if we do initiate a discussion, our analysis does not conclusively reveal whether the strategies will be effective in real cases (as mentioned in the Limitations), hence making it harder to clearly argue for and against any of these mitigation strategies. Having said that, we do discuss the value of the mitigation strategies in the second paragraph of the discussion and have made a minor clarification in response to feedback from another reviewer: “With regard to RQ2 the aim of this study was to provide insights into rebound effects of specific circular business models and the possible ways to mitigate them, that could be useful for future research as well as practitioners. Many mitigation strategies focus on influencing consumer actions through laws, incentives, nudging, price controls, or information campaigns ( Carrasco Campos, 2022; Kjaer et al., 2019; Laurenti et al., 2016; Sorrell et al., 2020). It is clear from these examples that circular businesses need consumer participation to transition from linear to circular consumption. However, there was little discussion on rebound effects linked to producer-producer business model relationships. And, while many rebound mitigation strategies were suggested, they were rarely empirically tested to assess their effectiveness and thus their actual effects remain theoretical. Indeed, only 32 of 45 papers linked mitigation strategies to specific rebound effects.”

**Comment 5.4:** Some measures mainly target high-income consumers (e.g., “Design products that feel expensive”, “Price control: encourage consumers to pay more for resource-efficient solutions”). These strategies raise issues of social equity and could even reinforce perceptions of injustice. It would be interesting to build on the already cited works (Mies & Gold, 2021; Schröder et al., 2019) to deepen this discussion.

**Response 5.4:** Thank you for this suggestion. We have now made the following additions to the discussion: “Lastly, past research calls for treating rebound effects as a risk to be managed in circular strategies ( Zerbino, 2022). Meanwhile, extensive studies have explored the circular economy's role in enhancing social sustainability and addressing the needs of disadvantaged communities ( Mies & Gold, 2021; Schröder et al., 2019). For example, some suggested mitigation strategies, e.g. price controls, suggest addressing rebound effects by making consumption more expensive. However, while raising prices may reduce consumption, it places disproportionate responsibility on lower-income groups, potentially exacerbating inequality (Mies & Gold, 2021; Schröder et al., 2019). Such approaches may also backfire if people simply switch to cheaper, more environmentally harmful products, and could fuel class-based resentment toward circular solutions perceived as accessible only to the wealthy. ”

**Comment 5.5:** Detailed Comments Page 9

1. “Conduct holistic assessment of the impacts to account for rebound effects”: Can assessment measures really be considered reduction strategies? This seems debatable.

Page 10

1. “Make efforts to change the lifestyles of consumers towards less impactful consumption”: please specify how this can be implemented.
2. “Use rewards and fines”: elaborate (what mechanisms, which responsible actors?).
3. “Prevent careless consumption” and “Inform consumers about the importance of product care”: how can these levers be concretely activated?
4. “Integrate multiple CE strategies within a business model”: why would this help reduce rebound effects?
5. “Eco-efficient value creation”: should be further detailed.
6. “Design to reduce the need for care”: wording is unclear.
7. “Introduction of a repairability index for products that can indicate a product’s durability”: what is the link with rebound effects?
8. “Introduce emission trading rules” and “Cap and trade schemes that place an upper limit on environmental stress”: clarify what these refer to and at what level of action they apply (corporate, public policy, etc.).

Page 12

1. “Prevent excessive consumption through sufficiency strategies”: please specify (what concrete strategies?).

**Response 5.5:** Thank you for these detailed comments. We would like to start by mentioning that we simply report the mitigation strategies suggested by the articles in our literature review sample, as mentioned at the end of Section 2.2:  “To maximize the traceability of the data the mitigation strategy descriptions have been kept as close to the source as possible, irrespective of their actual potential for the actual mitigation of rebound effects. The complete list of articles reviewed and the corresponding qualitative codes for rebound effects and mitigation strategies identified can be seen in Appendix A and C.” There are of course limitations related to this approach, and we reflect on this in detail in the Limitations section.

1. We would argue that it’s an essential part of managing rebound effects, since current practices rarely measure impacts of rebound effects, as one cannot reduce rebound effects if one does not assess the effects of the circular strategies one has implemented. In other words, it would be a management strategy, aimed at creating a feedback loop that supports and encourages the reduction and mitigation of rebound effects. However, as mentioned above, we are mainly reporting on the mitigation strategies suggested by articles in the sample.
2. The quoted suggestion is not elaborated upon in more detail in the literature that we have sampled and a more specific strategy proposal is as a result not possible. However, the essence of these strategies is encouraging sufficiency and to make less impactful consumption (i.e. consumption of goods and services with less environmental impact) more desirable and convenient. Although mentioned in the third paragraph of section 3.5 we have made the following elaboration:

“Other suggestions involved incentivising/promoting a change in the lifestyle of consumers towards less resource intensive products and services (for e.g., by making less impactful consumption more desirable or convenient or nudging towards more careful consumption) ( Vélez, 2023). Additional strategies included monitoring or forecasting the effects of the business model using LCA ( Abdoli et al., 2019; Alfarisi et al. 2023; Koide et al., 2022) or to be more efficient in value creation and use more recycled input materials ( Alfarisi et al. 2023).”

1. The responsible actor is specified in the table, where this particular strategy is listed as a strategy for businesses. Real-world examples already exist; for instance, the Dutch car-sharing company Greenwheels issues fines when a shared vehicle is returned in poor or dirty condition. We do not include this example in Table 4 since it is not part of our literature review sample, and our reporting follows the approach described in Section 2.2, where we keep mitigation strategies as close to the source material as possible. However, we do discuss it briefly in the second paragraph of Section 3.5.
2. The most commonly suggested methods are through nudging and incentives, i.e. reminders and encouraging better product care, as well as product care information that is easily digestible, or rewards for product care and fines for failure to perform adequate product care. This has been elaborated on in the third paragraph of Section 3.5. 
3. This is reported as it appears in the reviewed articles, following our approach of keeping mitigation strategies close to their source. The literature argues that combining strategies—such as reuse, repair, refurbishment, and efficiency—may help counterbalance rebound effects that could arise if only one strategy is used (e.g., efficiency gains triggering price-related rebounds). An example would be the introduction of sufficiency strategies to counteract the increased consumption that tends to occur when a product becomes more efficient. E.g. if your more efficient electric car - that you can afford to drive further - is actively encouraging you to utilise public transport, walk or travel by bicycle instead of driving. However, as mentioned previously, the reviewed studies do not provide empirical evidence on how this integration reduces rebounds, and we therefore highlight in the Limitations section that such strategies remain high-level and untested.
4. We have now changed this to: “Eco-efficient value creation (i.e. do more, with less)”
5. We have made the following addition: “Design to reduce the need for care, and making products more robust to different usage patterns”
6. A repairability index would allow consumers to more easily choose products that are more repair-friendly and have a longer lifespan. That way, the material flow of the product would be slowed. This was suggested by Beulque et al. (2023) in our sample to counter the short term  increase in sales that resulted from repair services offering discounts and vouchers when products were not repairable. Regarding the true effectiveness of this mitigation strategy, please see the explanation above related to reporting of the mitigation strategies.
7. The level of action is specified in the table as policy, i.e. these are a matter of public policy. 

# Reviewer report: Reviewer response for version 1

**DOI:** 10.21956/openreseurope.23046.r62331

**Published:** 2025-11-10

**Authors:** Vivian S.C Tunn

**Affiliations:** 1 Utrecht University, Utrecht, The Netherlands

This is a relevant and well written review of rebound effects in circular business models and related mitigation strategies. The paper provides a useful overview of rebound effects and mitigation strategies that have been identified and proposed in literature so far. The method is clearly described and the appendixes present more details on the literature review as well as the full list of reviewed articles. The article can be further improved, currently a definition of rebound effects is missing in the background section and a similar review with slightly different scope has been published in 2024 which means the contribution of this article should be rephrased. I provide more details on these points and additional comments below.

 Detailed comments:

1. In the introduction on p.4 (and also in the discussion) the authors state that “no systematic overview connects rebound effects to specific circular business model archetypes”, however, *Baczyk et al. (2024) Consumer behavior in circular business models: Unveiling conservation and rebound effects*, map which rebound mechanisms occur in different circular business models based on cases in literature they reviewed (see p.290).
2. On p.6 the authors state that others have misidentified unintended consequences as rebound effects. I suggest the authors add a definition of rebound effects and delineate rebound effects from unintended consequences to clarify their understanding of the concepts. Also regarding the definition of rebound effects, on p.17 the following is stated: “while some activities might appear to generate rebound effects, they might have long-term positive societal or natural restorative or regenerative effects”. In my understanding, even a circular business model with rebound effects can still be net positive unless the rebound effect is so large that it is a backfire. Reading the manuscript I wondered whether in the authors’ understanding rebound effects have to relate to emissions or if could also relate to other environmental or even social effects?
3. Great that the differences in samples between the two search strings are illustrated.
4. Large parts of the last paragraph of section 3.5. (p.14) are not clearly linked to PSS but general strategies to reduce rebound effects/ emissions.
5. Some of the business model archetypes should be more clearly defined and delineated. The business model archetypes “long life” and “next life” seem to overlap as both aim for extended product lifetimes and modular design seem implementations applicable to both. Please clarify the differences (is it about extended lifetime for the same user (long life) versus extended lifetime for different users (next life)?). Furthermore, on p.15 recycling is mentioned “Some studies have found that recycling can provide moral licensing to incentivize additional consumption”, clarify how this relates to the “next life” business model archetype.
6. On p.15 “circular efficiency measures” are mentioned that caused rebound effects, I suggest the authors specify which “circular efficiency measures” were implemented in the case study.
7. On p.16 it is stated that “past systematic reviews indicate limited awareness of rebounds”, specify which type of systematic reviews you are referring to (reviews of circular business models of business model innovation or something else?).

Minor comments:

- On p.7 the following sentences are either redundant or the reference to the mitigation sample is missing in the second sentence. “In the rebound sample the most common sector was mobility, followed by fashion and electrical appliances (Figure 3). The most studied sectors in the sample were mobility (19 studies) and electrical appliances (12).”
- The terms rebounds and rebound effects are used, are these synonymous or slightly different?
- Supplementary material, in Appendix C, I noticed that reference it should refer to Tunn & Ackermann 2020 instead of Ackermann & Tunn 2024.
- The terms business model strategy innovation and business model innovation are used, are these synonymous or slightly different?

- The following sentence on p.8 doesn’t flow quite right. “For example, one study by Walzberg et al. (2020) found that energy usage in a smart home that saved energy through automated temperature regulation, actually used more heating, reducing the amount of energy saved.” Consider removing “energy usage in” or rephrasing.
- One of the business model archetypes is inconsistently labeled. Once as “Detmaterialised or efficiency” as  by Pieroni et al. (2020) and other times as “Detmaterialised or sufficiency”. 

Are sufficient details of the methods and analysis provided to allow replication by others?

Yes

Are the rationale for, and objectives of, the Systematic Review clearly stated?

Yes

Is the statistical analysis and its interpretation appropriate?

Yes

Are the conclusions drawn adequately supported by the results presented in the review?

Yes

Reviewer Expertise:

Circular business models and rebound effects

I confirm that I have read this submission and believe that I have an appropriate level of expertise to confirm that it is of an acceptable scientific standard, however I have significant reservations, as outlined above.

## References

[1] : Consumer behavior in circular business models: Unveiling conservation and rebound effects. *Sustainable Production and Consumption* .2024;52: 10.1016/j.spc.2024.10.022 283-298 10.1016/j.spc.2024.10.022

# Response

**Published:** 2026-04-22

**Authors:** Ankita Das

**Affiliations:** Not Applicable, The Netherlands

Dear Reviewers,  Thank you for taking the time to review our article and provide your valuable feedback. We believe they have greatly improved the quality of the article. Please see our detailed responses below.

 Kind regards,

 The Authors

**Comment 4.1:** This is a relevant and well written review of rebound effects in circular business models and related mitigation strategies. The paper provides a useful overview of rebound effects and mitigation strategies that have been identified and proposed in literature so far. The method is clearly described and the appendixes present more details on the literature review as well as the full list of reviewed articles. The article can be further improved, currently a definition of rebound effects is missing in the background section and a similar review with slightly different scope has been published in 2024 which means the contribution of this article should be rephrased. I provide more details on these points and additional comments below. Detailed comments:

1. In the introduction on p.4 (and also in the discussion) the authors state that “no systematic overview connects rebound effects to specific circular business model archetypes”, however, Baczyk et al. (2024) Consumer behavior in circular business models: Unveiling conservation and rebound effects, map which rebound mechanisms occur in different circular business models based on cases in literature they reviewed (see p.290).

**Response 4.1:** Thank you for your comments! In line with your suggestion, and that of other reviewers, we now provide a clear definition of rebound effects at the start of the second paragraph of the introduction: “Despite the high potential of circular business models to reduce environmental impact ( Fidan et al., 2021; Kaddoura et al., 2019), rebound effects (REs) are increasingly recognized as a major challenge ( Andrew & Pigosso, 2024; Brockway et al., 2021; Zink & Geyer, 2017). Rebound effects are a form of negative systemic consequence that can offset or even increase the overall environmental impact (Zink & Geyer, 2017). While rebound effects can be both positive and negative (Font Vivanco et al., 2022; Zerbino, 2022), the focus of this paper is mainly on negative effects. Prominent examples include increases in consumption as a result of increased efficiency and lower prices (Chitnis et al., 2013), or increased wear and tear on shared products (Ackermann & Tunn, 2024). These effects arise from changes across business model elements, such as manufacturing and design choices (upstream), consumer behavior (downstream), and logistics (both upstream and downstream) ( Sarasini et al., 2024).” Furthermore, we have made the following changes to acknowledge the contribution of Baczyk et al. (2024): “Recent reviews by Lowe et al. (2024) and Zerbino (2022) developed methods to manage circular rebound effects, offering general guidelines but highlighting the lack of specific, actionable strategies for circular business models. Das et al. (2023) also attempted to classify rebounds across circular strategies through the Circular Rebound Tool, and a set of behavioral design strategies for preventing rebound effects have been consolidated (McKay & Pigosso, 2025). These studies emphasize the need for a more systematic analysis of rebound effects in circular business models and potential mitigation strategies. While past literature reviews exist, onlytwo articles connect rebound effects to specific circular business model archetypes or explore mitigation through business model design. In their review of case studies Baczyk et al. (2024) seek to clarify the role of consumer behaviour in shaping the effects of circular business model archetypes once implemented. They also give some suggestions for how rebound effects can perhaps be avoided, although these recommendations take the form of guiding principles and policy recommendations. Another recent contribution to rebound effect mitigation research is Mueller & Remke (2025). Focusing on mitigation through business model design, the authors developed the Circular Systems Sandbox, a business model design tool based on systems and life cycle thinking. The tool helps designers develop circular products and services and to understand the consequences their design choices may have on the environmental impact of their product or service. Although Circular Systems Sandbox connects business model choices to rebound effects, the focus is on the process of business model design, rather than archetype specific rebound effects and how one could mitigate them in practice. Clarifying the link between specific rebound effects and circular business model archetypes, as well as specific strategies for how to mitigate these rebound effects is an important first step toward integrating rebound mitigation into circular business model innovation, as empirical measurement is often impractical in early design phases.”

**Comment 4.2**:

1. On p.6 the authors state that others have misidentified unintended consequences as rebound effects. I suggest the authors add a definition of rebound effects and delineate rebound effects from unintended consequences to clarify their understanding of the concepts. Also regarding the definition of rebound effects, on p.17 the following is stated: “while some activities might appear to generate rebound effects, they might have long-term positive societal or natural restorative or regenerative effects”. In my understanding, even a circular business model with rebound effects can still be net positive unless the rebound effect is so large that it is a backfire. Reading the manuscript I wondered whether in the authors’ understanding rebound effects have to relate to emissions or if could also relate to other environmental or even social effects?

**Response 4.2:** Thank you for your comment! Although rebound effects are differentiated from unintended consequences in the discussion: “Additionally, the literature sometimes used "rebound effects" more broadly to describe unintended consequences of circular business models. While rebound effects typically refer to negative outcomes linked to market dynamics and consumer behavior, unintended consequences encompass broader, unforeseen impacts of implementing such models. Business decision-makers may not consciously distinguish between induced impacts, unintended consequences, and rebound effects. In at least 13 papers from the initial sample, this distinction was unclear. We argue that "rebound effects" should specifically refer to systemic or behavioral responses triggered by adopting a circular business model, that are not essential elements that are part of its implementation ( Kjaer et al., 2018).” We agree that it would be appropriate to properly define rebound effects in the introduction. We have therefore made the following changes to the second paragraph of the introduction:  “Despite the high potential of circular business models to reduce environmental impact ( Fidan et al., 2021; Kaddoura et al., 2019), rebound effects (REs) are increasingly recognized as a major challenge that can offset or even increase overall environmental impact ( Andrew & Pigosso, 2024; Brockway et al., 2021; Zink & Geyer, 2017). Rebound effects are a form of systemic consequence that can offset or even increase the overall environmental impact (Zink & Geyer, 2017).  While rebound effects can be both positive and negative (Font Vivanco et al., 2022; Zerbino, 2022), the focus of this paper is mainly on negative effects. Prominent examples include increases in consumption as a result of increased efficiency and lower prices (Chitnis et al., 2013), or increased wear and tear on shared products (Ackermann & Tunn, 2024).  These effects arise from changes across business model elements, such as manufacturing and design choices (upstream), consumer behavior (downstream), and logistics (both upstream and downstream) ( Sarasini et al., 2024).” You are quite correct that a circular business model with rebound effects can still be a net positive - indeed there are several studies that show net positive effects in spite of rebound effects. Research has shown that the circular economy has played a role in enhancing social sustainability (Font Vivanco et al., 2022). We therefore argue that it could at times be important to see beyond measures such as CO2-emissions, and perhaps even accept some rebound effects as part of achieving or maintaining socially sustainable gains. One concrete example is poorer households receiving thermal retrofits (Font Vivanco et al., 2022). This would produce rebound effects, but is also incredibly important to help end energy poverty and provide people with dignified living.

**Comment 4.3:**

1. Great that the differences in samples between the two search strings are illustrated.
2. Large parts of the last paragraph of section 3.5. (p.14) are not clearly linked to PSS but general strategies to reduce rebound effects/ emissions.

**Response 4.3:** Thank you for your comment! Although these might seem like general suggestions, they were in fact either specifically proposed as strategies to counter rebound effects on PSS or were proposed in papers that examined the size of rebound effects in PSS. In other words, they are in the right paragraph. We have made further reference to this in our limitations, where we comment on the many of the proposed mitigation strategies being very general: “Third, while several mitigation strategies were identified, many were broad suggestions rather than actionable steps (for e.g., in the case of PSS, price controls and steering consumers towards less impactful consumption or measuring impacts of change). ”

**Comment 4.4:**

1. Some of the business model archetypes should be more clearly defined and delineated. The business model archetypes “long life” and “next life” seem to overlap as both aim for extended product lifetimes and modular design seem implementations applicable to both. Please clarify the differences (is it about extended lifetime for the same user (long life) versus extended lifetime for different users (next life)?). Furthermore, on p.15 recycling is mentioned “Some studies have found that recycling can provide moral licensing to incentivize additional consumption”, clarify how this relates to the “next life” business model archetype.

**Response 4.4: **Thank you for your comment! There can indeed appear to be some overlap between these two circular business model archetypes. Long life refers to strategies that extend the life of a product and retain their value, so as to avoid premature disposal, which would represent a waste. Next life, meanwhile, refers to strategies that seek to capture value at the end of the use phase, and these strategies include recycling, reusing or repurposing products, such as through second hand sales. We have made the following changes in Sections 3.6 and 3.7 in order to provide examples of business model strategies associated with each archetypal cluster: “The archetype cluster “long life" refers to business models focused on extended product lifetimes for the same user often facilitated by design for attachment or timeless design, durability, and high levels of warranties and service, coupled with a premium pricing model ” “The circular business model archetype cluster “next life” refers to business models that help extend the lifetime of products and to slow resource loops. While long life aims to extend product lif time for a single user, next life aims to extend the material or product lifetime beyond the single user. This can be facilitated by strategies such as design for dis-and reassembly, upgradability, maintenance and repair, as well as product take-back models and second hand platforms ” “Some studies have found that recycling can provide moral licensing to incentivize additional consumption, since consumers can view the recycled alternatives as more eco-friendly and thus end up using more of the products, increasing overall consumption related impacts.”

**Comment 4.5:**

1. On p.15 “circular efficiency measures” are mentioned that caused rebound effects, I suggest the authors specify which “circular efficiency measures” were implemented in the case study.
2. On p.16 it is stated that “past systematic reviews indicate limited awareness of rebounds”, specify which type of systematic reviews you are referring to (reviews of circular business models of business model innovation or something else?).

**Response 4.5: **Thank you for your comments! The circular efficiency measures that pertained to the study by Konash & Nasr (2022) have been specified as per your suggestion:  “In the case of strategies that aim to increase efficiency or reduce consumption, some studies found that these strategies can result in direct or indirect rebounds. For example, Konash & Nasr (2022) describe a direct rebound in a case study of a medium sized complex parts manufacturing company from New York, USA. They found that the company's circular efficiency measures, such as equipment upgrades, energy and water use monitoring, optimizing operating schedules, recycling water and materials, reducing waste, performance improvement programs, actually led to increased resource use in the short term and a clear backfire effect.” Thank you for this comment! We have made the following changes in line with your comment: “Past systematic reviews of circular economy rebounds indicate limited awareness of the topic in practice ( Lowe et al., 2024; Zerbino, 2022). In their review of rebound estimation methods Lowe et al. (2024) found that beyond tax and pricing policies, discussions on mitigating rebounds are scarce, especially in a circular economy context.”

**Comment 4.6: **Minor comments:

- On p.7 the following sentences are either redundant or the reference to the mitigation sample is missing in the second sentence. “In the rebound sample the most common sector was mobility, followed by fashion and electrical appliances (Figure 3). The most studied sectors in the sample were mobility (19 studies) and electrical appliances (12).”

**Response 4.6:** Thank you for noticing this error. We have rectified this error by removing the second sentence now.

**Comment 4.7:**

- The terms rebounds and rebound effects are used, are these synonymous or slightly different?
- Supplementary material, in Appendix C, I noticed that reference it should refer to Tunn & Ackermann 2020 instead of Ackermann & Tunn 2024.
- The terms business model strategy innovation and business model innovation are used, are these synonymous or slightly different?

**Response 4.7:** Thank you for your comments!

1. Yes, these are synonymous. We have clarified this in the 4th paragraph of the introduction:

“ The study uses the term rebound effects (or simply rebounds) instead of circular rebound effects, as many rebound mechanisms apply to circularity as well. ”

1. Thank you, we have corrected the reference!
2. Yes these terms are synonymous, we have now standardised them to ‘business model innovation’

**Comment 4.8:**

- The following sentence on p.8 doesn’t flow quite right. “For example, one study by Walzberg et al. (2020) found that energy usage in a smart home that saved energy through automated temperature regulation, actually used more heating, reducing the amount of energy saved.” Consider removing “energy usage in” or rephrasing.
- One of the business model archetypes is inconsistently labeled. Once as “Detmaterialised or efficiency” as  by Pieroni et al. (2020) and other times as “Detmaterialised or sufficiency”. 

**Response 4.8:** Thank you for your comments! 1. Yes, it is quite long, containing more than one dependent clause. We have made the following changes in the hope that it will flow better:  “For example, one study by Walzberg et al. (2020) found that energy usage in a smart home - that saved energy through automated temperature regulation - actually used more heating, reducing the amount of energy saved.” 2. Thank you for noticing this error. This inconsistency has been corrected now.

# Reviewer report: Reviewer response for version 1

**DOI:** 10.21956/openreseurope.23046.r62333

**Published:** 2025-11-08

**Authors:** Christian Koldewey, Julia-Marie Vehmeyer

**Affiliations:** 1 Paderborn University, Paderborn, Germany

Dear authors,

 Thank you for the opportunity to review your interesting paper.

 The article addresses a highly relevant issue: rebound effects in circular business models and strategies for mitigating them. This topic is important both scientifically and practically, as rebound effects often diminish the positive environmental contribution of the circular economy. However, they have not been systematically investigated yet. The authors provide a valuable synthesis of existing empirical studies and offer an initial structured link between circular business model archetypes and rebound mechanisms.

 Overall, the article is well organized and transparent, with a sound and well-documented methodological approach. Nonetheless, some areas would benefit from clarification and refinement, as outlined below.

**Introduction**

 The introduction is clearly written, and the research questions are well developed. The research gap is convincingly justified, and the linkage to existing work is strong. The authors cite their prior works extensively; however, since Pigosso and Bocken are leading scholars in the field, this appears justified, however the focus should remain on seminal and relevant contributions. Vague statements like “Similarly, Pigosso (2024) called for advancing design science to incorporate rebound effects into business model design.” should be reduced or specified.

 Furthermore, while the elaborations on rebound effects are comprehensive and interesting, a clear definition of this central concept for the paper is missing. The paper would greatly benefit from an explicit definition to establish conceptual clarity for the remainder of the paper.

**Method**

 The research method is described in detailed and with transparency. However, the section begins by blending the *state of the art* and the *justification for the chosen method*. For clarity, the delineation to Lowe et al. and Zerbino should be made in the introduction and the method chapter should explain why a systematic literature review is suitable to answer the research question.

 There are two main issues concerning the search strategy:

**First**, the search strings themselves contain inconsistencies. In Search String 1, the terms “sustainable business”, “circular business*”, and “circular*” are linked with OR. “circular*” already includes “circular business*”, making the expression partly redundant, while “sustainable business” makes the query overly broad because it captures much non-circular literature. *Search String 2* introduces slightly different terms (“circular business model*” instead of “circular business*”) and (“sustainab*” instead of “sustainable business”) without justification. The rationale behind these changes is unclear and should be clarified.

**Second**, the connection between the search strings and the research questions could be improved. *Research Question 2* focuses on strategies to mitigate rebound effects, while *Search String 2* mainly targets environmental impacts. Thus, its logic does not fully match the intent of the second question. Either the research question or the string should be refined for consistency.

 While the inclusion of multiple synonyms for “rebound effects” is commendable and ensures comprehensiveness, the search strings would benefit from greater conceptual coherence and clearer alignment with the stated research question. In general, the authors should reflect and argue on their ambitions regarding comprehensiveness and precision.

 The inclusion criteria are otherwise sound, and the use of archetypal clusters (Pieroni et al., 2020) and the distinction between direct, indirect, and economy-wide rebound effects are clearly explained. A minor issue concerns the cut-off date (September 2024); more recent publications may already exist.

**Results**

 The results section is comprehensive and clearly structured, providing a detailed overview of the analyzed studies and their categorization according to circular business model archetypes. The descriptive characteristics of the reviewed studies are well presented and transparent. The authors identify gaps (e.g., geographic concentration, methodological limitations), and these are sufficiently described.

 The results for each archetype are presented clearly, and the accompanying tables effectively support the textual discussion. However, several of the proposed mitigation strategies, such as “urban planning to reduce travel time” are general sustainability policies rather than rebound-specific measures. A clearer distinction between genuinely circular and generic interventions would improve precision.

 Regarding the “Product-service systems” archetype, the definition provided in the paper (“combinations of products and services such as rental and lease”) is too narrow and does not fully reflect Tukker’s (2004) conceptualization. Tukker distinguishes eight PSS types. Beyond rental and leasing, PSS also includes models such as product-related services, consultancy (e.g., for the most efficient use) and functional result-oriented services, which differ in ownership, risk, and sustainability potential. The results of the SLR mainly address rebound effects in use-oriented models (e.g., renting, leasing, pay-per-use).  The authors should either expand their definition of PSS accordingly and include relevant rebound effects or clarify why rebound effects were only observed for this subgroup and possibly re-examine the data for other PSS types.

**Discussion**

 The discussion is generally well-developed. It would profit from a stronger interpretative stance on the results (e.g., by suggesting categories of mitigation strategies) to be less repetitive and descriptive. The numbering in 4.1 should be removed and a stronger focus on answering the RQs explicitly should be given. In the section on limitations (4.3), the authors might explicitly mention review-specific constraints, such as reliance on only two databases and the potential for omission during title or full-text screening.

**Conclusion**

 The conclusion provides a clear and concise summary of the study’s main insights and contributions. It highlights the relevance of linking circular business model archetypes with rebound mechanisms and offers valuable guidance for future research and practice. Overall, it closes the paper effectively and leaves the reader with a coherent understanding of the study’s contribution.

**Minor issues**

 There are a few minor issues throughout the paper, such as typographical errors and inconsistent spacing. Careful proofreading is recommended.

**Overall assessment**

 The paper is a valuable and thorough contribution to the growing literature on circular business models and rebound effects. Its main strengths lie in its systematic approach, clarity, and relevance. The main areas for improvement concern methodological precision (alignment of research questions and search strings), refinement of the archetype discussion, and a clearer distinction between rebound-specific and general sustainability measures.

 Hopefully, these comments will help you further strengthen this already solid and insightful paper.

Are sufficient details of the methods and analysis provided to allow replication by others?

Partly

Are the rationale for, and objectives of, the Systematic Review clearly stated?

Yes

Is the statistical analysis and its interpretation appropriate?

Yes

Are the conclusions drawn adequately supported by the results presented in the review?

Yes

Reviewer Expertise:

Circular Business Models, Circular Product Design

We confirm that we have read this submission and believe that we have an appropriate level of expertise to confirm that it is of an acceptable scientific standard, however we have significant reservations, as outlined above.

# Response

**Published:** 2026-04-22

**Authors:** Ankita Das

**Affiliations:** Not Applicable, The Netherlands

Dear Reviewers,  Thank you for taking the time to review our article and provide your valuable feedback. We believe they have greatly improved the quality of the article. Please see our detailed responses below.

 Kind regards,

 The Authors 

**Comment 3.1:** Dear authors, Thank you for the opportunity to review your interesting paper. The article addresses a highly relevant issue: rebound effects in circular business models and strategies for mitigating them. This topic is important both scientifically and practically, as rebound effects often diminish the positive environmental contribution of the circular economy. However, they have not been systematically investigated yet. The authors provide a valuable synthesis of existing empirical studies and offer an initial structured link between circular business model archetypes and rebound mechanisms. Overall, the article is well organized and transparent, with a sound and well-documented methodological approach. Nonetheless, some areas would benefit from clarification and refinement, as outlined below. Introduction The introduction is clearly written, and the research questions are well developed. The research gap is convincingly justified, and the linkage to existing work is strong. The authors cite their prior works extensively; however, since Pigosso and Bocken are leading scholars in the field, this appears justified, however the focus should remain on seminal and relevant contributions. Vague statements like “Similarly, Pigosso (2024) called for advancing design science to incorporate rebound effects into business model design.” should be reduced or specified. Furthermore, while the elaborations on rebound effects are comprehensive and interesting, a clear definition of this central concept for the paper is missing. The paper would greatly benefit from an explicit definition to establish conceptual clarity for the remainder of the paper.

**Response 3.1: **Thank you for taking the time to review our paper and provide your insightful comments! We have made the following changes to the Introduction to reduce vague statements: “Despite the high potential of circular business models to reduce environmental impact ( Fidan et al., 2021; Kaddoura et al., 2019), rebound effects (REs) are increasingly recognized as a major challenge ( Andrew & Pigosso, 2024; Brockway et al., 2021; Zink & Geyer, 2017). Rebound effects are a form of negative systemic consequence that can offset or even increase the overall environmental impact (Zink & Geyer, 2017). While rebound effects can be both positive and negative (Font Vivanco et al., 2022; Zerbino, 2022), the focus of this paper is mainly on negative effects. Prominent examples include increases in consumption as a result of increased efficiency and lower prices (Chitnis et al., 2013), or increased wear and tear on shared products (Ackermann & Tunn, 2024). These effects arise from changes across business model elements, such as manufacturing and design choices (upstream), consumer behavior (downstream), and logistics (both upstream and downstream) ( Sarasini et al., 2024).”

**Comment 3.2: **Method The research method is described in detailed and with transparency. However, the section begins by blending the state of the art and the justification for the chosen method. For clarity, the delineation to Lowe et al. and Zerbino should be made in the introduction and the method chapter should explain why a systematic literature review is suitable to answer the research question.

**Response 3.2: **Thank you for your comment. We have made the following change to the start of section 2: “A systematic literature review ( Bryman & Bell, 2011) was conducted to address the research questions,  give an overview over the state of the art as well as a direction for future research. This method was chosen to allow a detailed investigation of rebound effects and their mitigation in the context of circular business model archetypes or business model design.”

**Comment 3.3:** There are two main issues concerning the search strategy: First, the search strings themselves contain inconsistencies. In Search String 1, the terms “sustainable business”, “circular business*”, and “circular*” are linked with OR. “circular*” already includes “circular business*”, making the expression partly redundant, while “sustainable business” makes the query overly broad because it captures much non-circular literature. Search String 2 introduces slightly different terms (“circular business model*” instead of “circular business*”) and (“sustainab*” instead of “sustainable business”) without justification. The rationale behind these changes is unclear and should be clarified. Second, the connection between the search strings and the research questions could be improved. Research Question 2 focuses on strategies to mitigate rebound effects, while Search String 2 mainly targets environmental impacts. Thus, its logic does not fully match the intent of the second question. Either the research question or the string should be refined for consistency. While the inclusion of multiple synonyms for “rebound effects” is commendable and ensures comprehensiveness, the search strings would benefit from greater conceptual coherence and clearer alignment with the stated research question. In general, the authors should reflect and argue on their ambitions regarding comprehensiveness and precision. The inclusion criteria are otherwise sound, and the use of archetypal clusters (Pieroni et al., 2020) and the distinction between direct, indirect, and economy-wide rebound effects are clearly explained. A minor issue concerns the cut-off date (September 2024); more recent publications may already exist.

**Response 3.3: **Thank you for your comment! The reason for using overlapping and broad search terms was to ensure we sufficiently captured relevant literature on the topic.  Although both search strings are numbered S-1 and S-2 are not meant to correspond to R1 and R2 respectively. Rather S-1 and S-2 were used as complementary searches to ensure a comprehensive search of the topic. Subsequently the title and abstract scans were used to identify papers that could answer R1 and/or R2. With the title and abstract scan including 1091 papers it's fair to say that the search prioritised comprehensiveness rather than precision. We realise the previous framing might have been misleading, and have added the following changes to Section 2.1 to clarify this further: “The primary reason for including two search strings was that while circular business models is a relatively new term, concepts that are included within the term now  have existed much longer, such as efficiency and concepts within sustainability, of which circular business models can be said to be subset (Pieroni et al., 2019, 2020). Further, it was initially found that a focus on just circular and sustainable business models might be too narrow to capture all research available on their related rebound effects. However, broadening the synonyms to include the terms “business model*” and “business*” in S-2 captured too many irrelevant studies, and so this search string was further focused to specifically include environmental impacts.” We have also added the following sentence to the first paragraph of the limitations: “It is also worth noting that the search was carried out in late 2024 and for that reason it does not encompass all the more recent contributions to the field.”

**Comment 3.4**: Results The results section is comprehensive and clearly structured, providing a detailed overview of the analyzed studies and their categorization according to circular business model archetypes. The descriptive characteristics of the reviewed studies are well presented and transparent. The authors identify gaps (e.g., geographic concentration, methodological limitations), and these are sufficiently described. The results for each archetype are presented clearly, and the accompanying tables effectively support the textual discussion. However, several of the proposed mitigation strategies, such as “urban planning to reduce travel time” are general sustainability policies rather than rebound-specific measures. A clearer distinction between genuinely circular and generic interventions would improve precision.

**Response 3.4:** Thank you for your encouraging comment. We agree that some of the suggested mitigation strategies that we found in the literature are quite general and not always specific to circular business models or their rebound effects. In section 4.3 of the discussion this is mentioned as a limitation of the present study, but we have made the following additions in line with your reflections: “Third, while several mitigation strategies were identified, many were broad suggestions rather than actionable steps (for e.g., in the case of PSS, price controls and steering consumers towards less impactful consumption or measuring impacts of change). While others referred to initiatives that themselves can also lead to rebound effects (e.g., suggesting to improve the social perception of secondhand goods, practice that itself is connected to several rebound effects). Further, some of the suggested policy-oriented strategies, such as urban planning, fuel or carbon tax and restrictions on fossil fuel transportation are not specific to the circular economy, and could lead to additional rebound effects if not carefully planned and implemented. Moreover, most strategies were not empirically tested, meaning their actual effectiveness remains uncertain. This study, therefore, relies on mitigation strategies as reported by authors, highlighting the need for future research to identify credible, context-specific strategies and quantitatively assess their effectiveness.”

**Comment 3.5: **Regarding the “Product-service systems” archetype, the definition provided in the paper (“combinations of products and services such as rental and lease”) is too narrow and does not fully reflect Tukker’s (2004) conceptualization. Tukker distinguishes eight PSS types. Beyond rental and leasing, PSS also includes models such as product-related services, consultancy (e.g., for the most efficient use) and functional result-oriented services, which differ in ownership, risk, and sustainability potential. The results of the SLR mainly address rebound effects in use-oriented models (e.g., renting, leasing, pay-per-use).  The authors should either expand their definition of PSS accordingly and include relevant rebound effects or clarify why rebound effects were only observed for this subgroup and possibly re-examine the data for other PSS types.

**Response 3.5:** Thank you for your suggestion. You are correct in your summation of PSS according to Tukker (2004), however, and that the initial description in Section 3.5 was too brief. We base our definition upon the categorisation defined by Pieroni et al. (2020), hence the slightly narrower focus on what PSS models are. We did provide a broader definition in Table 4 “(Product-Service Systems (E.g., Product-as-a- service, Rental, Hire, Leasing, Pay-per-use, functional sales)” previously, but for clarity we have added this definition in Section 3.5: “The archetype cluster of “product-service systems” serves as an umbrella term for combinations of products and services such as product-as-a- service, rental, hire, leasing, pay-per-use and functional sales (Pieroni et al., 2020; Tukker, 2004), that aim to reduce consumption by offering products as services.”

**Comment 3.6:** Discussion The discussion is generally well-developed. It would profit from a stronger interpretative stance on the results (e.g., by suggesting categories of mitigation strategies) to be less repetitive and descriptive. The numbering in 4.1 should be removed and a stronger focus on answering the RQs explicitly should be given. In the section on limitations (4.3), the authors might explicitly mention review-specific constraints, such as reliance on only two databases and the potential for omission during title or full-text screening.

**Response 3.6:** Thank you for your comments and insights! We have made changes to section 4.1 to make the links to the research questions clearer, and removed the numbering. Regarding the suggestions for changes to the limitations section, we have added the following sentences to clarify the limitations of the method better: “This study had some limitations. First, there are limitations pertaining to the chosen method. The literature search was carried out in only two databases, this might have led to search results being more limited than they otherwise might have been. Additionally, although the title, abstract and full text scans were carried out systematically, there is a chance for inadvertent omissions of relevant data, due to human error.”

**Comment 3.7: **Conclusion The conclusion provides a clear and concise summary of the study’s main insights and contributions. It highlights the relevance of linking circular business model archetypes with rebound mechanisms and offers valuable guidance for future research and practice. Overall, it closes the paper effectively and leaves the reader with a coherent understanding of the study’s contribution. Minor issues There are a few minor issues throughout the paper, such as typographical errors and inconsistent spacing. Careful proofreading is recommended. Overall assessment The paper is a valuable and thorough contribution to the growing literature on circular business models and rebound effects. Its main strengths lie in its systematic approach, clarity, and relevance. The main areas for improvement concern methodological precision (alignment of research questions and search strings), refinement of the archetype discussion, and a clearer distinction between rebound-specific and general sustainability measures. Hopefully, these comments will help you further strengthen this already solid and insightful paper.

**Response 3.7: **Thank you for all your kind words and for your insightful comments! We have carried out diligent proofreading as suggested.

# Reviewer report: Reviewer response for version 1

**DOI:** 10.21956/openreseurope.23046.r62330

**Published:** 2025-11-06

**Authors:** Mariantonietta Ferrante

**Affiliations:** 1 Politecnico di Bari, Bari, Italy

The topic of this paper is highly relevant and makes a significant contribution to the field of research. The study's focus on the relationship between circular business models and rebound effects is particularly compelling. The authors conduct a systematic literature review and provide a comprehensive summary of rebound effects, organized across seven different circular business model archetypes. The paper also clearly explains how rebound effects can manifest within these circular business model archetypes, along with the associated strategies for mitigation. The inclusion of tables throughout the paper significantly aids in clarifying the work and enhancing its readability.

 The background section is well-written and effectively sets the stage for the study, outlining the rationale behind the research. The objective of the paper is clearly defined and logically structured.

 One point for improvement would be to ensure consistency in the use of acronyms throughout the paper. I suggest reviewing the document to confirm that all acronyms are introduced and defined at their first occurrence.

 The methodology section is well-structured and presented in a clear, coherent manner. Figure 1 is particularly useful in illustrating the research methodology, and the tables presenting the search protocol and criteria used to select relevant documents are a helpful addition.

 The authors present their results effectively, and the results section is generally well-written and easy to follow. However, Table 4 is quite lengthy and may hinder the paper's readability. It could be split into smaller tables, perhaps one for each archetype, with the content condensed to improve clarity.

 The discussion section is clear, and the authors provide useful comparisons with other works in the field. However, the use of lists in this section may be somewhat distracting and could be streamlined for better readability. While the contributions are well outlined, they could be made more specific by linking the circular business model archetypes directly to the rebound effects and offering more practical insights on how these effects could be mitigated.

 The conclusion provides a suitable summary of the paper's key findings and contributions. However, the section discussing limitations and future developments would be better placed at the end of the conclusion for better structural flow.

Are sufficient details of the methods and analysis provided to allow replication by others?

Yes

Are the rationale for, and objectives of, the Systematic Review clearly stated?

Yes

Is the statistical analysis and its interpretation appropriate?

Yes

Are the conclusions drawn adequately supported by the results presented in the review?

Partly

Reviewer Expertise:

Circular economy, Circular manufacturing, Rebound effects

I confirm that I have read this submission and believe that I have an appropriate level of expertise to confirm that it is of an acceptable scientific standard, however I have significant reservations, as outlined above.

# Response

**Published:** 2026-04-22

**Authors:** Ankita Das

**Affiliations:** Not Applicable, The Netherlands

Dear Reviewers,  Thank you for taking the time to review our article and provide your valuable feedback. We believe they have greatly improved the quality of the article. Please see our detailed responses below. Kind regards,

 The Authors --

**Comment 2.1:** The topic of this paper is highly relevant and makes a significant contribution to the field of research. The study's focus on the relationship between circular business models and rebound effects is particularly compelling. The authors conduct a systematic literature review and provide a comprehensive summary of rebound effects, organized across seven different circular business model archetypes. The paper also clearly explains how rebound effects can manifest within these circular business model archetypes, along with the associated strategies for mitigation. The inclusion of tables throughout the paper significantly aids in clarifying the work and enhancing its readability. The background section is well-written and effectively sets the stage for the study, outlining the rationale behind the research. The objective of the paper is clearly defined and logically structured. One point for improvement would be to ensure consistency in the use of acronyms throughout the paper. I suggest reviewing the document to confirm that all acronyms are introduced and defined at their first occurrence.

**Response 2.1: **Thank you for taking the time to review our paper, and for the positive review. We have reviewed the paper in line with your suggestion and added acronyms where necessary.

**Comment 2.2:** The methodology section is well-structured and presented in a clear, coherent manner. Figure 1 is particularly useful in illustrating the research methodology, and the tables presenting the search protocol and criteria used to select relevant documents are a helpful addition. The authors present their results effectively, and the results section is generally well-written and easy to follow. However, Table 4 is quite lengthy and may hinder the paper's readability. It could be split into smaller tables, perhaps one for each archetype, with the content condensed to improve clarity.

**Response 2.2: **Thank you for your kind comment! You’re quite right that Table 4 is very large and contains a fair amount of detail. At the same time, this table allows us to summarize information that will be difficult to contain in the main text, and the information in the table has already been condensed to a bare minimum. Meanwhile, we have also had requests from reviewers to add more detail to the results section, rather than further condense the information that’s presented. We have found it challenging to balance the different suggestions that have been made for the results section. As for splitting the table into several tables, we tried this, but have been concerned that that will make the article difficult to navigate, given the amount of size any of these 7 tables would need to occupy (most of a page). We did not find that this improves readability. Given the diverse opinions on this topic we have chosen to keep the table as is. 

**Comment 2.3: **The discussion section is clear, and the authors provide useful comparisons with other works in the field. However, the use of lists in this section may be somewhat distracting and could be streamlined for better readability. While the contributions are well outlined, they could be made more specific by linking the circular business model archetypes directly to the rebound effects and offering more practical insights on how these effects could be mitigated.

**Response 2.3:** Thank you for your comment! It may however be difficult to offer further practical insight into how specific rebound effects can be mitigated, since one of the main findings of the review is that the suggested mitigation strategies that were found have yet to be properly tested and evaluated. We reflect on this further in the fifth paragraph of Section 4.1 and in the limitations section in 4.3. In this sense, the practical insight is that while there are many suggestions about mitigating rebound effects, it is yet unknown if these ideas can be translated into successful rebound effect mitigation.

**Comment 2.4:** The conclusion provides a suitable summary of the paper's key findings and contributions. However, the section discussing limitations and future developments would be better placed at the end of the conclusion for better structural flow.

**Response 2.4: **Thank you for this suggestion! We have however elected to keep the conclusion shorter, without subsections to enable clearer readability of the findings for non-academic readers who benefit from this research.

# Reviewer report: Reviewer response for version 1

**DOI:** 10.21956/openreseurope.23046.r62332

**Published:** 2025-10-28

**Authors:** Konstantin Remke

**Affiliations:** 1 Strategy & Entrepreneurship, Ecole de Management de Normandie, Clichy, Paris, Normandy, France; 2 ESCP Business School, Paris, France

**Summary and General Remarks:**

 Thank you for giving me the opportunity to review the manuscript "Rebound effects in circular business models: A review of cases and mitigation strategies" to be published in ORE. The manuscript reviews the extant literature on circular economy rebound effects and idenfies direct and indirect effects for different business model archetypes. Additionally, the manuscript extracts circular economy rebound effect mitigation strategies that have been described by extant literature. 

 The manuscript offers an important, novel, and interesting account of circular economy rebound effects and mitigation strategies. The manuscript's research design works well with the outset of the study, and the findings yield interesting insights.

 While the manuscript topic-wise seems promising, in the following I would like to point to some avenues for improvement:

**Introduction:**

 - In the first paragraph, the authors describe which conversation they join. However, at this point they are mainly engaged in citing their own articles. While some of these articles may certainly relate and are central to the conversation at hand, others do not really relate (e.g., business model experimentation; Bocken et al., 2021b). Although the first paragraph appears to mainly serve the explanation of circular business models, referring specifically to the experimentation aspect feels forced. In contrast, a more specific explanation of what circular business model (archetypes) are would have been useful. Despite later being central to the method, the circular business model archetypes categorization by Pieroni et al. (2020) is not even mentioned in the introduction.

 - In the second paragraph, the authors then open up on the conversation around rebound effects and the challenges they pose for circular business models. However, the authors overlook articles important for providing the full picture of the conversation and that are well aligned with the argument that "business model design can strongly influence product care and sustainability performance" when referring to PSS, to finally mitigate circular economy rebound effects (e.g., Mueller & Remke, 2025 - ref 2; Siderius & Poldner, 2021 - ref 1).

 - In the third paragraph, the description of circular economy rebound remains a bit shallow given the fact that the manuscript does not include a theoretical background section. Instead of just mentioning more recent studies in one sentence, these could have been described in more detail to provide the full picture of how the circular economy rebound effect concept evolved.

 - In the fourth and fifth paragraphs, the authors describe circular economy rebound effect mitigation strategies and highlight articles that approached these. Furthermore, the authors argue that more design science is needed for advancing circular economy rebound effect mitigation literature such as the Circular Economy Rebound Tool by Das et al. (2023). They conclude that "no systematic overview connects rebound effects to specific business model archetypes or explores mitigation through business model designs". It is very surprising that the authors are not mentioning or referring to the article by Mueller and Remke (2025) (or alternatively Remke & Mueller, 2024 - ref 3) (throughout their entire manuscript), which does exactly what the authors describe as being a gap in extant literature (apart from conducting a systematic literature review). The article applies design science to develop and even test a tool that is targeted at circular economy rebound mitigation through linking circular product design, PSS design, business model design (and explicitly archetypes), and circular ecosystem design. Thus, a part of the underlying research gap of the manuscript to be reviewed here, has basically already been filled. At least, the authors should have been aware of at least one of the two publications. Thus, the contribution "this is a critical first step toward integrating rebound mitigation into design science" is not an actual contribution.

 - The abstract and introductions would benefit from including more detailed findings from the literature review. Just referring to "the findings revealed an apparent lack of quantitative research on mitigation strategies" is too narrow.

**Method:**

 - It remains a bit unclear what a literature review has to do with design science (as initially claimed to be part of the contribution in the introduction). This could have been better argued in the first paragraph of the method section. Maybe the reason for this is a different understanding of what is meant by design science in your manuscript. Thus, this concept would need further clarification. To check upon established research on what design science is, you may refer to for example Dimov et al. (2023) - ref 4.

 - While using the Pieroni et al. (2020) circular business model archetypes is fair, this again may be understood as being self-promotional. Why did the authors not use a different classification? The argument that Pieroni et al. would be the "most comprehensive classification" is a bit shallow. Please provide a more sophisticated argument on this.

 - The authors do not explain the journal selection criteria. This is problematic because it influences the quality of the reviewed literature sample. Upon checking the reviewed articles, the reviewed papers are mainly published in lower-tier journals. This is undermining the overall quality and contributions of the manuscript.

**Results:**

 - The results could have been a bit more detailed. For some circular business model categories, the authors only provide one example for why it may cause circular economy rebound effects. As this is the most insightful part of the manuscript, it should be more elaborated. The same applies for the mitigation strategies, which could be explained in greater detail (for now one sentence on average).

**Discussion:**

 - The discussion should follow a clearer structure of theoretical contributions and practical implications. In the current version (especially the first part of the discussion), it is difficult to follow for the reader as the argumentation is not optimally interwoven with the manuscript's findings. 

 - The articles cited at the very beginning of the introduction (e.g., business model experimentation) are not really mentioned further in the discussion, confirming the impressions mentioned for the introduction section. 

 - The theoretical contributions do not yet connect well with the actual conversation on circular economy rebound mitigation. However, I feel that there could be a decent contribution to the conversation upon optimized argumentation and connection with extant literature on that topic.

 - In the introduction, the authors are claiming to making a contribution to design science in the context of circular economy rebound mitigation. However, this claim is not picked up in the discussion. 

 - The practical implications could be described in greater detail.

**Conclusion:**

 - The conclusion is overly repetitive and can be condensed to the essence of the final paragraph.

**Minor Comments:**

 - There are some writing and punctuation errors.

 - The authors continuously provide "roadmaps" at the beginning of each section. This could be avoided by a clearer flow of argumentation as it takes up a lot of space.

Are sufficient details of the methods and analysis provided to allow replication by others?

Partly

Are the rationale for, and objectives of, the Systematic Review clearly stated?

Yes

Is the statistical analysis and its interpretation appropriate?

Partly

Are the conclusions drawn adequately supported by the results presented in the review?

Partly

Reviewer Expertise:

Circular economy, circular business model innovation, circular economy rebound mitigation, circular systems design, design science research

I confirm that I have read this submission and believe that I have an appropriate level of expertise to confirm that it is of an acceptable scientific standard, however I have significant reservations, as outlined above.

## References

[1] : Reconsidering the Circular Economy Rebound effect: Propositions from a case study of the Dutch Circular Textile Valley. *Journal of Cleaner Production* .2021;293: 10.1016/j.jclepro.2021.125996 10.1016/j.jclepro.2021.125996

[2] : Circular Systems Sandbox: A process tool for circular systems design and rebound effect reduction. *Journal of Business Venturing Design* .2025;4: 10.1016/j.jbvd.2025.100028 10.1016/j.jbvd.2025.100028

[3] : Overcoming Rebound Effects: A Process Blueprint for Circular Systems Design.14621: 10.1007/978-3-031-61175-9_3 33-47 10.1007/978-3-031-61175-9_3

[4] : Crafting and Assessing Design Science Research for Entrepreneurship. *Entrepreneurship Theory and Practice* .2023;47(5) : 10.1177/10422587221128271 1543-1567 10.1177/10422587221128271

# Response

**Published:** 2026-04-22

**Authors:** Ankita Das

**Affiliations:** Not Applicable, The Netherlands

Dear Reviewers,  Thank you for taking the time to review our article and provide your valuable feedback. We believe they have greatly improved the quality of the article.

 Please see our detailed responses below. Kind regards,

 The Authors --  

**Comment 1.1 **Summary and General Remarks: 

 Thank you for giving me the opportunity to review the manuscript "Rebound effects in circular business models: A review of cases and mitigation strategies" to be published in ORE. The manuscript reviews the extant literature on circular economy rebound effects and idenfies direct and indirect effects for different business model archetypes. Additionally, the manuscript extracts circular economy rebound effect mitigation strategies that have been described by extant literature.  The manuscript offers an important, novel, and interesting account of circular economy rebound effects and mitigation strategies. The manuscript's research design works well with the outset of the study, and the findings yield interesting insights. While the manuscript topic-wise seems promising, in the following I would like to point to some avenues for improvement:

 Introduction: - In the first paragraph, the authors describe which conversation they join. However, at this point they are mainly engaged in citing their own articles. While some of these articles may certainly relate and are central to the conversation at hand, others do not really relate (e.g., business model experimentation; Bocken et al., 2021b). Although the first paragraph appears to mainly serve the explanation of circular business models, referring specifically to the experimentation aspect feels forced. In contrast, a more specific explanation of what circular business model (archetypes) are would have been useful. Despite later being central to the method, the circular business model archetypes categorization by Pieroni et al. (2020) is not even mentioned in the introduction.

**Response 1.1: **Thank you for your kind words and for your comment!  We added the Pieroni et al. (2020) paper now earlier on in the paper, although our original intention was to introduce it later. Business model experimentation was an important starting point for our research as we noticed that rebound effects can be mitigated through good design (see also Das, A., Konietzko, J., Bocken, N., & Dijk, M. (2023). The Circular Rebound Tool: A tool to move companies towards more sustainable circular business models. Resources, conservation & recycling advances, 20, 200185). Hence, we kept this focus/ rationale in the introduction but improved the opening paragraph based on your suggestions. As a result of this comment, we have made the following changes in line with this comment:  “Circular business models (CBMs) aim to maximize value by slowing, closing, narrowing and/or regenerating material flows to extend resource use and minimize waste ( Konietzko et al., 2020). The implementation of CBMs often involve a number of business model patterns and archetypes, both upstream and downstream (Pieroni et al., 2020; Rosa et al., 2019; Salvador et al., 2023). While companies experiment with new circular business models to test their desirability, feasibility, and viability, their performance in terms of circularity and sustainability is rarely tested ( Bocken et al., 2021a, b). Companies usually rely on rules of thumb to forecast impact (i.e., heuristics related to circular/sustainable design such as guidelines and policies), which can be inaccurate ( Das et al., 2022). Negative side effects of circular business models can potentially be prevented during the design and experimentation phase so understanding these is essential (Das et al., 2023; Pigosso, 2024). Past research has found that negative side effects of circular business models can potentially be prevented during the design and experimentation phase so understanding these is essential (Das et al., 2023).”

**Comment 1.2: **- In the second paragraph, the authors then open up on the conversation around rebound effects and the challenges they pose for circular business models. However, the authors overlook articles important for providing the full picture of the conversation and that are well aligned with the argument that "business model design can strongly influence product care and sustainability performance" when referring to PSS, to finally mitigate circular economy rebound effects (e.g., Mueller & Remke, 2025 - ref 2; Siderius & Poldner, 2021 - ref 1). - In the third paragraph, the description of circular economy rebound remains a bit shallow given the fact that the manuscript does not include a theoretical background section. Instead of just mentioning more recent studies in one sentence, these could have been described in more detail to provide the full picture of how the circular economy rebound effect concept evolved.  

**Response 1.2: **Thank you for your comment! We have made the following changes to the second paragraph: “ Managerial decisions in the early design phase can have a big impact on the sustainability performance of a product ( McAloone & Bey, 2009). Pigosso (2024) argues that rebound effects are not yet integrated into the business model design phase and should be proactively addressed by embedding awareness and mitigation strategies from the outset. This aligns with research on product-service systems, which highlights how business model design can strongly influence product care ( Ackermann & Tunn, 2024; Müller& Remke, 2025) and sustainability performance (Siderius & Poldner, 2021; Tukker, 2015), indicating the importance of business model design in managing rebound effects.” We also made the following changes to the fourth (previously third) paragraph, in line with your comments: “More recent research has identified several further mechanisms through which rebounds can occur and provided a more detailed causal understanding of how rebound effects work ( Castro et al., 2022; Guzzo et al., 2024; Lange et al., 2021). This ranges from economic to behavioral and social practice mechanisms, at both the consumer and producer sides; directly, indirectly, through changes in demand and use intensity on different levels of the economy (on the level of the individual firm, the sector, economy wide or global), and at different points in time (at implementation, in response to implementation or at the point of attempting to mitigate rebounds) (Aguiar et al., 2026; Andrew et al., 2026; Ferrante et al., 2026; Van Der Loo & Pigosso, 2024). This study uses the term rebound effects (or simply rebounds) instead of circular rebound effects, as many rebound mechanisms apply to circularity as well. An example would be in the classic case of fuel-efficient cars leading to users driving them for longer distances ( Pardi, 2021). Or, in the case of PSS, it has been found that rented items tend to be treated less carefully since consumers feel less attached and bear no long-term responsibility, leading to higher replacement rates ( Ackermann & Tunn, 2024; Schaefers et al., 2016). This illustrates a link between business model design and rebound effects (McKay et al., 2025).”  

**Comment 1.3:** - In the fourth and fifth paragraphs, the authors describe circular economy rebound effect mitigation strategies and highlight articles that approached these. Furthermore, the authors argue that more design science is needed for advancing circular economy rebound effect mitigation literature such as the Circular Economy Rebound Tool by Das et al. (2023). They conclude that "no systematic overview connects rebound effects to specific business model archetypes or explores mitigation through business model designs". It is very surprising that the authors are not mentioning or referring to the article by Mueller and Remke (2025) (or alternatively Remke & Mueller, 2024 - ref 3) (throughout their entire manuscript), which does exactly what the authors describe as being a gap in extant literature (apart from conducting a systematic literature review). The article applies design science to develop and even test a tool that is targeted at circular economy rebound mitigation through linking circular product design, PSS design, business model design (and explicitly archetypes), and circular ecosystem design. Thus, a part of the underlying research gap of the manuscript to be reviewed here, has basically already been filled. At least, the authors should have been aware of at least one of the two publications. Thus, the contribution "this is a critical first step toward integrating rebound mitigation into design science" is not an actual contribution. - The abstract and introductions would benefit from including more detailed findings from the literature review. Just referring to "the findings revealed an apparent lack of quantitative research on mitigation strategies" is too narrow.

**Response 1.3: **Thank you for your comments. We have made the added the following changes to the Introduction:  “While past literature reviews exist, only two articles connect rebound effects to specific circular business model archetypes or explore mitigation through business model design. In their review of case studies Baczyk et al. (2024) seek to clarify the role of consumer behaviour in shaping the effects of circular business model archetypes once implemented. They also give some suggestions for how rebound effects can perhaps be avoided, although these recommendations take the form of guiding principles and policy recommendations. Another recent contribution to rebound effect mitigation research is Mueller & Remke (2025). Focusing on mitigation through business model design, the authors developed the Circular Systems Sandbox, a business model design tool based on systems and life cycle thinking. The tool helps designers develop circular products and services and to understand the consequences their design choices may have on the environmental impact of their product or service. Although Circular Systems Sandbox connects business model choices to rebound effects, the focus is on the process of business model design, rather than archetype specific rebound effects and how one could mitigate them in practice. Clarifying the link between specific rebound effects and circular business model archetypes, as well as specific strategies for how to mitigate these rebound effects is an important first step toward integrating rebound mitigation into circular business model innovation, as empirical measurement is often impractical in early design phases. Thus, this paper aims to identify empirically measured rebound effects in circular business models and potential mitigation strategies, addressing the following research questions:” As pertains to the lack of quantitative research on mitigation strategies, this refers to the result of our systematic overview. As elaborated in the discussion, while some papers did quantitatively investigate the size of rebound effects, no papers were found that actually provided a quantitative measurement of the effect of rebound mitigation strategies that had been implemented. To clarify this we have made the following changes to the discussion: “And, while many rebound mitigation strategies were suggested, they were rarely empirically tested to assess their effectiveness and thus their actual effects remain theoretical. Indeed, only 32 of 45 papers linked mitigation strategies to specific rebound effects. Additionally, some suggested strategies were overly general and could unintentionally reinforce rebound mechanisms, increasing environmental impact.”

**Comment 1.4: **Method: - It remains a bit unclear what a literature review has to do with design science (as initially claimed to be part of the contribution in the introduction). This could have been better argued in the first paragraph of the method section. Maybe the reason for this is a different understanding of what is meant by design science in your manuscript. Thus, this concept would need further clarification. To check upon established research on what design science is, you may refer to for example Dimov et al. (2023) - ref 4. - While using the Pieroni et al. (2020) circular business model archetypes is fair, this again may be understood as being self-promotional. Why did the authors not use a different classification? The argument that Pieroni et al. would be the "most comprehensive classification" is a bit shallow. Please provide a more sophisticated argument on this. - The authors do not explain the journal selection criteria. This is problematic because it influences the quality of the reviewed literature sample. Upon checking the reviewed articles, the reviewed papers are mainly published in lower-tier journals. This is undermining the overall quality and contributions of the manuscript.

**Response 1.4:** Thank you for your comment. We have now removed references to design science research to make the contributions more streamlined. Regarding your point about the Pieroni et al. (2020) paper, it was the first author of the present paper (not a co-author of the Pieroni et al 2020 work) who selected this paper initially. The main reason for this was the upstream/ downstream categorization. We removed any ‘promotional-sounding’ text and focused on the categorization instead. It should be noted that this list of archetypes is based on an extensive consolidation and analysis of 180+ CEBM cases, and is well-cited,  thus having a strong empirical base for the work. So this was an additional reason for seeing this as a valid approach.  Regarding the journal selection criteria: we recognise that circular business model literature is widespread and interdisciplinary so we deliberately chose to keep the sample broad to capture the widest possible sample of studies (including upcoming and early stage research) that empirically and quantitatively measure rebound effects, since at the time of conducting the initial systematic literature review there were no other studies published that had conducted such a meta-analysis. 

**Comment 1.5: **Results: - The results could have been a bit more detailed. For some circular business model categories, the authors only provide one example for why it may cause circular economy rebound effects. As this is the most insightful part of the manuscript, it should be more elaborated. The same applies for the mitigation strategies, which could be explained in greater detail (for now one sentence on average).

**Response 1.5: **Thank you for this comment. The rebound effects and mitigation strategies reported in the table are entirely based on the results of the systematic literature review, and the results quantified and reported by the articles in the sample. Hence, some circular business model categories have less rebound effects reported compared to others. We have chosen to stick strictly to only the results reported by the articles in our sample.  One of our challenges was to find ways to present as much detail as possible, whilst still making the results very readable. We therefore chose to collect a full account of the results in table 4 and to provide narrative summary in the text. 

**Comment 1.6: **Discussion:  - The discussion should follow a clearer structure of theoretical contributions and practical implications. In the current version (especially the first part of the discussion), it is difficult to follow for the reader as the argumentation is not optimally interwoven with the manuscript's findings.  - The theoretical contributions do not yet connect well with the actual conversation on circular economy rebound mitigation. However, I feel that there could be a decent contribution to the conversation upon optimized argumentation and connection with extant literature on that topic.

**Response 1.6: **Thank you for your comment. A more detailed discussion of the contributions of the study to research, practice and policy has already been included in Section 4.2, and the Discussion section has now undergone significant revision in line with the comments received from the peer review.

**Comment 1.7: **- The articles cited at the very beginning of the introduction (e.g., business model experimentation) are not really mentioned further in the discussion, confirming the impressions mentioned for the introduction section.  - In the introduction, the authors are claiming to making a contribution to design science in the context of circular economy rebound mitigation. However, this claim is not picked up in the discussion.  - The practical implications could be described in greater detail.

**Response 1.7:** Thank you for your comments! You’re quite correct that Linder & Williander, 2017, Bocken et al., 2021a and Bocken et al., 2021b are not cited in the discussion. That said, the link with business model experimentation is firmly made in the third and fourth paragraphs of section 4.1,  with relevant citations, as well as in the second paragraph of section 4.2:  “Further, this paper aims to provide insights into rebound effects in specific circular business models and potential mitigation strategies useful for research and practice. Organizing rebound effects by circular business model archetypes, assuming they are shaped by the model implemented, offers clearer information on risks and mitigation strategies applicable to specific businesses. Similarly, categorizing rebound effects as direct, indirect, or economy-wide helps assess how innovations impact different economic levels and interact. Identifying the system in which a rebound effect occurs may better enable practitioners and policymakers to influence environmental impacts. Additionally, the systematic overview can help determine which rebound effects and mitigation strategies fall within zone of influence. Finally, such an overview may offer valuable guidance in early business model design, where companies often rely on “rules of thumb” rather than detailed assessments to estimate environmental impacts ( Das et al., 2022; Das et al., 2023).”  And in section 4.2, where the implication of our systematic overview for practitioners is discussed in terms of the different phases of business model experimentation:  “For practitioners the results provide suggestions for mitigation strategies that can be trialed and refined. Innovators can use these insights to identify and address potential rebound effects early in business model design, reducing risk—especially in light of potential new regulations. Designers can also consult the overview during the early ideation phase of business model design ( Bocken & Snihur, 2020) to assess and mitigate rebound effects. Further, in the experimentation phase, the systematic overview can continue to be a reference for identifying rebound effects and refining mitigation strategies as business model performance is evaluated and adjusted.” We have now also removed references to design science research in the Introduction to make our contributions more streamlined.  Although the discussion of the practical implications in section 4.1 can benefit from some further detail, we are wary of repetition, given that the practical implications are discussed in detail in section 4.2. That said, in service of readability and to make the discussion of the practical implications more salient, have made the following changes to the third paragraph of section 4.1: “Additionally, the systematic overview can help determine which rebound effects and mitigation strategies fall within the zone of influence of practitioners, hopefully further aiding them in developing strategies to avoid or mitigate rebound effects. Finally, such an overview may offer valuable guidance in early business model design, where companies often rely on “rules of thumb” rather than detailed assessments to estimate environmental impacts ( Das et al., 2022; Das et al., 2023).”

**Comment 1.8:** Conclusion: - The conclusion is overly repetitive and can be condensed to the essence of the final paragraph. Minor Comments:  - There are some writing and punctuation errors. - The authors continuously provide "roadmaps" at the beginning of each section. This could be avoided by a clearer flow of argumentation as it takes up a lot of space.

**Response 1.8: **Thank you for your comment! We have made the following changes to the conclusion to make it more concise: “While research on circular rebound effects has grown, there has been a lack of organized knowledge on mitigating these effects within business models. This study aimed to investigate (1) the potential rebound effects of circular business model archetypes and (2) identified strategies for their mitigation through a systematic literature review. It provides a structured overview mapping rebound effects across seven circular business model archetypal clusters, and suggestions for ways to potentially mitigate them. These findings help bridge a critical gap in circular economy and business model innovation literature, offering academics and practitioners a structured way to consider the true net environmental impact of new circular product and business model ideas, and preventing rebound effects that could undermine their benefits. The study highlights where empirical research has focused on direct, indirect, and economy-wide rebound effects and where future research opportunities exist. It also provides policymakers and practitioners with a systematic overview of risks associated with circular business models and potential mitigation strategies. However, while mitigation strategies have been suggested, they remain largely untested, and their effectiveness is uncertain. The results indicate the need for more research to attribute rebound effects to specific circular business model choices, standardise methods to analyze the behavior and magnitude of these rebound effects, and develop mitigation strategies. Additional research could also refine the key findings of this paper (Table 4) into a practical tool or process that could enhance their usability. Finally, while mitigating rebounds is crucial, their occurrence must be weighed against the broader societal benefits of circular business models.” Thank you for your comment regarding the punctuation errors, we have now read through the document and made the necessary changes. Further regarding the comment on the ‘roadmaps’, while we appreciate the suggestion, we provided these in order to improve readability, and make the paper more accessible to readers outside of academia, such as business leaders and innovators, who constitute an important audience for this research. Thus, after careful consideration we have chosen to leave them as is.
